# ROCK inhibitors upregulate the neuroprotective Parkin-mediated mitophagy pathway

**DOI:** 10.1038/s41467-019-13781-3

**Published:** 2020-01-03

**Authors:** Natalia Moskal, Victoria Riccio, Mikhail Bashkurov, Rediet Taddese, Alessandro Datti, Peter N. Lewis, G. Angus McQuibban

**Affiliations:** 1 Department of Biochemistry, University of Toronto, Toronto, ON Canada; 2 Network Biology Collaborative Centre, Mount Sinai Hospital, Toronto, ON Canada; 3 Department of Agriculture, Food, and Environmental Sciences, University of Perugia, Perugia, Italy

**Keywords:** Small molecules, Parkinson's disease

## Abstract

The accumulation of damaged mitochondria causes the death of dopaminergic neurons. The Parkin-mediated mitophagy pathway functions to remove these mitochondria from cells. Targeting this pathway represents a therapeutic strategy for several neurodegenerative diseases, most notably Parkinson’s disease. We describe a discovery pipeline to identify small molecules that increase Parkin recruitment to damaged mitochondria and ensuing mitophagy. We show that ROCK inhibitors promote the activity of this pathway by increasing the recruitment of HK2, a positive regulator of Parkin, to mitochondria. This leads to the increased targeting of mitochondria to lysosomes and removal of damaged mitochondria from cells. Furthermore, ROCK inhibitors demonstrate neuroprotective effects in flies subjected to paraquat, a parkinsonian toxin that induces mitochondrial damage. Importantly, parkin and rok are required for these effects, revealing a signaling axis which controls Parkin-mediated mitophagy that may be exploited for the development of Parkinson’s disease therapeutics.

## Abstract (web summary)

Damaged mitochondria are known to cause neuronal death, suggesting clearance as a potential therapy. Here, Moskal et al. show that ROCK inhibitors promote Parkin recruitment and mitophagy and have neuroprotective effects in fruit flies challenged with a toxin that induces mitochondrial damage.

## Introduction

The survival of dopaminergic neurons depends on the ability to mitigate mitochondrial damage. Mutations in the genes which encode PINK1 and Parkin, two key mediators of a common pathway which targets damaged mitochondria for degradation, cause early-onset Parkinson’s disease (PD)^1,2^. Moreover, dysfunction in these proteins has also been observed in sporadic PD^3,4^. By amplifying the ability to degrade damaged mitochondria, it may be possible to prevent the degeneration of dopaminergic neurons and to delay disease progression. Indeed, the amplification of PINK1 activity using the neo-substrate kinetin triphosphate (KTP) improves the survival of dopaminergic neurons challenged with oxidative stress^5^.

We sought to identify small-molecule potentiators of Parkin, the E3 ubiquitin ligase downstream of PINK1 (ref. ^6^). Following mitochondrial damage, PINK1 is stabilized on the outer mitochondrial membrane^7^. Several PINK1-mediated phosphorylation events lead to recruitment of Parkin from the cytosol to the mitochondria^8,9^. Parkin then designates substrates for proteasomal or autophagic degradation, by tagging them with ubiquitin.

Several RNAi-based screens have quantified the recruitment of Parkin to damaged mitochondria to identify upstream modulators of Parkin-mediated mitophagy^10–12^. These efforts have led to the identification of several regulators of Parkin activity including TOMM7, HspA1L, BAG4, SIAH3, ATPIF1 and HK2. Instead, we sought to identify small molecules which modified the Parkin recruitment step in the mitophagy cascade.

A screen of ~3000 compounds led to the identification of several Parkin activators with a common canonical target: Rho-associated protein kinase (ROCK). Several previous studies demonstrated the protective effect of ROCK inhibition in disease models of neurodegeneration, including PD (for a review, see Koch et al.^13^). However, the role of ROCK in the Parkin-mediated mitophagy pathway has been unexplored. We present evidence that ROCK may be exploited as a molecular switch for Parkin-mediated mitophagy. We use inhibitors of ROCK to augment Parkin-mediated mitophagy, ultimately leading to improvements of PD-related phenotypes in vivo.

## Results

### Screen for small-molecule modulators of Parkin-mediated mitophagy

We developed a high-throughput small-molecule screen aimed at identifying molecules that increase the proportion of cells with Parkin localized to mitochondria upon induction of mitophagy with the protonophore, carbonyl cyanide m-chlorophenylhydrazone (CCCP) (Supplementary Fig. 1). This subcellular transition is impaired in several PD-linked Parkin mutants, resulting in the impaired clearance of outer mitochondrial membrane (OMM) substrates and the reduced turnover of damaged mitochondria (Supplementary Fig. 2)^14,15^. Likewise, RNAi targeting upstream modulators of Parkin activity visibly affects this step in the mitophagy cascade. Consequently, we conducted a screen aimed at identifying molecules that increased Parkin recruitment to damaged mitochondria.

We created and screened HEK293 cell lines stably expressing GFP-tagged Parkin. The protein kinase inhibitor (*n* = 480) and Prestwick libraries (*n* = 1120) were screened in these cells across two independent replicates. Our three-day screening protocol consisted of (1) seeding cells, (2) pinning small molecules at 4 µM concentration and (3) inducing mitophagy with CCCP, proceeded by processing of plates for high content imaging (Supplementary Fig. 3).

Supervised machine learning was used to classify cells into two classes: (1) cells with cytosolic Parkin distribution and (2) those with Parkin localized to mitochondria. The percentage of cells with mitochondrial Parkin in wells pinned with DMSO, instead of small molecule, prior to CCCP addition was 83.4 ± 2.3% and 71.2 ± 2.6% for the kinase inhibitor and Prestwick library screens, respectively. The average percentage of cells with mitochondrial Parkin in negative control wells, untreated with CCCP, was 3.7 ± 2.6% and 1.1 ± 1.1% for the kinase inhibitor and Prestwick library screens, respectively, indicating good separation between negative and positive controls.

### Several ROCK inhibitors increase Parkin recruitment to damaged mitochondria

**Fig. 1:**
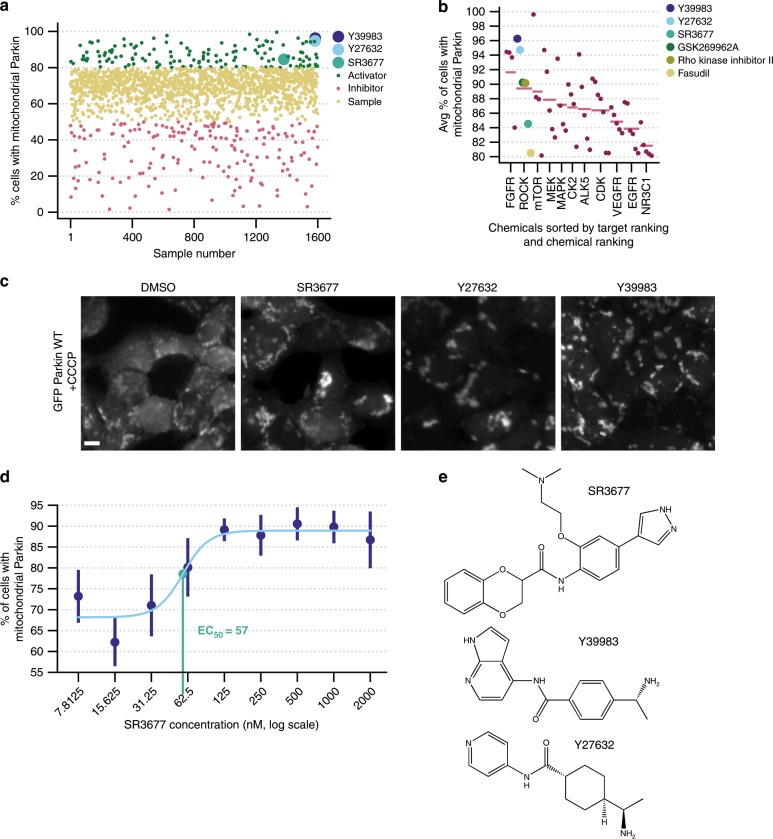
ROCK inhibitors increase Parkin recruitment to damaged mitochondria. **a** Average activity (% of cells with mitochondrial Parkin distribution) of all compounds screened. Based on these values, compounds are classified as either activators (green), samples (yellow) or inhibitors (pink). ROCK inhibitors Y27632 (light blue), Y39983 (navy) and SR3677 (light green) are highlighted. **b** Enhancers of Parkin recruitment from **a** arranged according to common protein target. ROCK inhibitors Fasudil (yellow), rho kinase inhibitor II (olive) and GSK2699662A (green). Pink dashes represent average value amongst each group and pink dots represent activity values for activator compounds whose canonical target is not ROCK. **c** GFP Parkin distribution in cells following incubation in 4 µM of small-molecule library compound for 16 h and mitophagy induction with 2 h of 20 µM CCCP treatment (*n* = 2 independent experiments). Scale bars, 10 µm. **d** HEK293 GFP Parkin cells were treated with varying doses of SR3677 for 2 h prior to induction of mitophagy with 10 µM CCCP for 1 h. Dose response curves were fitted to these data, yielding an EC_50_ value of 57 nM. Data are expressed as mean ± s.e.m (*n* = 3 independent experiments). Error bars represent s.e.m. **e** Chemical structures of ROCK inhibitors identified.

Small molecules which yielded average values >80% were considered candidate hits, while those yielding average values below 50% were considered inhibitors (Fig. 1a). Staurosporine, a known activator of Parkin-mediated mitophagy^16,17^, was recovered in the top 0.3% of hits, indicating the capability of the screen to identify true mitophagy activators. To maximize our odds of recovering true positives^18^, we arranged Parkin recruitment activators according to their protein target (Fig. 1b). Inhibitors of FGFR, ROCK and MEK had the highest average Parkin recruitment values. Of these three, the ROCK inhibitor series was the largest recovered, with six ROCK inhibitors identified as activators. The average percentage of cells with mitochondrial Parkin amongst these six compounds is 89.4%, in the kinase inhibitor screen where the positive controls average 71.2%. Another compelling reason to follow up on this family of inhibitors is the evidence in the literature for autophagy enhancement and improvement of phenotypes associated with neurodegeneration following treatment with these compounds^19,20^.

For follow-up, we selected the two highest ranking ROCK inhibitors, Y27632 and Y39983, in addition to a more selective inhibitor of ROCK2, SR3677 (Fig. 1d)^21^. ROCK2 is enriched in brain tissue, so selectively targeting this isoform may minimize the unnecessary inhibition of ROCK in non-neuronal tissues^22^. Principle component analysis was performed on Morgan fingerprints corresponding to each activator to evaluate chemical similarity amongst the hits^18^. Besides the common protein target of these Parkin recruitment activators, the three hits selected for further validation share the greatest degree of structural similarities (Supplementary Fig. 4).

Y27632 and Y39983 are tool compounds frequently employed to modulate ROCK activity. These compounds inhibit both ROCK1 and ROCK2 in biochemical and cell-based assays (for a review, see ref. ^23^); however, due to poor cell permeability, they are administered at high concentrations in cell-based assays ranging from 10 to 100 µM^20,24^. On the other hand, the IC_50_ of SR3677 for ROCK2, the isoform enriched in the nervous system, is lower (3 nM) than for ROCK1 (56 nM). The low IC_50_ and greater selectivity of SR3677 for ROCK2 may confer therapeutic advantages^25^.

Surprisingly, SR3677 ranked lower than the other ROCK inhibitors in our kinase inhibitor library screen despite its lower IC_50_ in cell-based assays^25^. So, we re-tested the effect of varying doses of SR3677 on Parkin recruitment to determine the optimal concentration to use in subsequent experiments (Fig. 1c). By 0.5 µM, the maximal effect of SR3677 was achieved and increased Parkin recruitment to damaged mitochondria was observed following long (17 h) and short (2 h) pre-incubation (Fig. 1a, c; Supplementary Fig. 5a). Importantly, Parkin distribution was diffuse in cells treated with SR3677 in the absence of CCCP (Supplementary Fig. 5b). Parkin recruitment activators which cause mitochondrial damage (Supplementary Fig. 6)^26,27^ recruit Parkin in the absence of CCCP.

### Identification of inhibitors of Parkin recruitment to damaged mitochondria

In addition to enhancers of Parkin recruitment to damaged mitochondria, the screen identified several inhibitors of Parkin recruitment to damaged mitochondria. By elucidating the mechanism of action of these molecules, we may identify previously uncharacterized regulators of the Parkin-mediated mitophagy pathway, which may guide the development of therapeutics in the future. Parkin-mediated mitophagy inhibitors may be tested in PD model systems which exhibit excessive mitophagy, such as PD-causing mutations W403A in *PARK2* and A53T in *PARK1* (ref. ^28^).

The Parkin recruitment inhibitor families identified in our screen include compounds targeting FLT3, EGFR, MET, CDK, JAK, checkpoint (CHK) and Aurora (AURK) kinases, in addition to prostaglandin synthase (PTGS) and tubulin (TUB) (Supplementary Fig. 7). Both Aurora (AURK) and cyclin-dependent (CDK) kinases promote Drp1 activity and its mitochondrial recruitment, which are both prerequisites for stabilization of PINK1 on the outer mitochondrial membrane^29,30^.

One group of Parkin recruitment inhibitors identified were FLT3 inhibitors, such as Ac220 (Supplementary Fig. 7). Upon retesting, Ac220 inhibited Parkin recruitment and degradation of one of its outer mitochondrial membrane substrates, Mfn2 (Supplementary Figs. 8, 9a, c)^31^. Mfn2 degradation is critical for driving mitophagy forward by facilitating the segregation of damaged mitochondria from the healthy mitochondrial network and the dissociation between the ER and the mitochondria^32,33^ Following Ac220 treatment, PINK1 fails to accumulate in response to mitochondrial damage (Supplementary Fig. 9b, d, e).

Parkin recruitment and Mfn2 degradation were inhibited by Ac220 in a dose-dependent manner (Supplementary Figs. 8b, 9c). Currently, the most frequently employed methods to inhibit mitophagy consist of blocking lysosomal acidification (bafilomycin or chloroquine) or general autophagy (PI3K inhibitors)^17^. Specific inhibitors of this pathway may serve as valuable chemical tools. Additionally, inhibition of Parkin-mediated mitophagy has been shown to sensitize drug-resistant cancer cells to chemotherapy which presents an alternate therapeutic application^34^.

### ROCK inhibitors enhance mitophagy at several steps in the mitophagy cascade

**Fig. 2:**
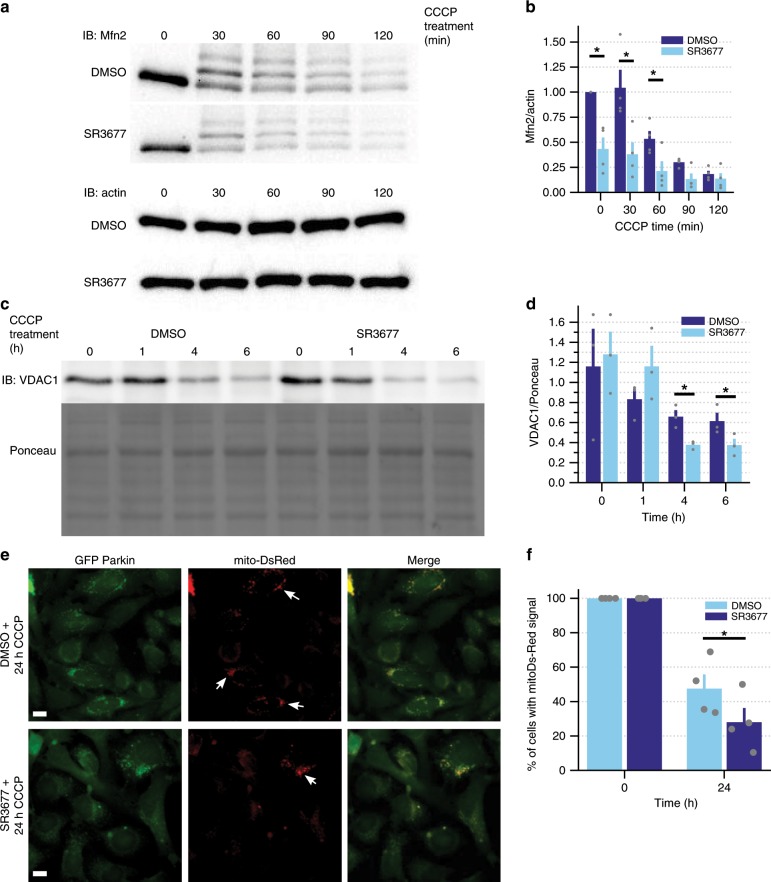
SR3677 reduces mitochondrial mass upon induction of mitochondrial damage. HEK293 GFP Parkin cells treated with 0.5 µM SR3677 or DMSO for 2 h were incubated with 10 µM CCCP for the indicated time in minutes or hours. Cell lysates were harvested, proteins were separated by SDS-PAGE and immunoblotting was performed with **a** anti-Mfn2, anti-actin and **c** anti-VDAC1 antibodies. Ponceau staining was performed prior to immunoblotting as a loading control. **b**, **d** Densitometry analysis was performed to quantify Mfn2 (**a**) and VDAC1 (**c**) levels in each sample, followed by normalization to actin loading control (**a**) (*n* = 4 independent experiments), or Ponceau staining (**c**) (*n* = 3 independent experiments). Data are expressed as mean ± s.e.m. *P*-values were determined by paired one-tailed Student’s *t*-test, *P < 0.05. **e** HeLa cells stably expressing mito-DsRed and GFP Parkin were pre-treated with either DMSO or 0.5 µM SR3677, followed by 24-h treatment with 10 µM CCCP or DMSO. Scale bars, 10 µm. **f** Quantification of the percentage of cells that retained mitochondrial (mito-DsRed) signal. Cells with positive mitochondrial signal are indicated by arrows in **e**. Data are expressed as mean ± s.e.m (*n* = 4 independent experiments). *P*-values were determined by paired one-tailed Student’s *t*-test, *P < 0.05. Error bars represent s.e.m.

In contrast to Ac220, 2/3 ROCK inhibitors (Y39983, SR3677) enhanced Mfn2 degradation (Fig. 2a, b; Supplementary Fig. 10). However, in order to achieve comparable increases to Mfn2 degradation, Y39983 had to be administered at 10 µM, while SR3677 could significantly enhance Mfn2 degradation at 0.5 µM, a 20-fold lower concentration. The poor cell permeability of the amino-pyridine series of ROCK inhibitors likely accounts for this difference in working concentration. Due to this difference in potency and its greater selectivity for the ROCK isoform that is enriched in neurons, we elected to focus subsequent validation experiments on SR3677.

Consistently, SR3677 increased the turnover of another outer mitochondrial membrane Parkin substrate, VDAC1 (refs. ^31,35^) (Fig. 2c, d). We also examined the degradation of proteins residing in other submitochondrial compartments. SR3677 increased the degradation of inner mitochondrial membrane proteins ATP5A and COXIV (Supplementary Fig. 22a, c). Likewise, the degradation of UQCRC2, a matrix-facing subunit of the IMM protein COXIII, was enhanced by SR3677 co-treatment (Supplementary Fig. 22b). Both UQCRC2 and ATP5A are represented within Parkin’s ubiquitylome^35^.

We also quantified mitochondrial mass in HeLa cells expressing GFP-Parkin and mito-DsRed, where DsRed is targeted to the mitochondrial matrix. Previous studies have found that prolonged depolarization leads to complete loss of mito-DsRed signal in a large proportion of cells^36^, so we quantified the percentage of cells which retained mito-DsRed signal to assess mito-DsRed clearance. While both DMSO- and SR3677-treated cells retained mitochondrial mass following 24 h of DMSO treatment, 47.5 ± 8.25% of cells pre-treated with DMSO retained mito-DsRed following 24 h of CCCP treatment, compared to only 28 ± 8.21% of cells pre-treated with SR3677 (Fig. 2f, g). These orthogonal methods show that SR3677 enhances degradation of proteins in various submitochondrial compartments.

**Fig. 3:**
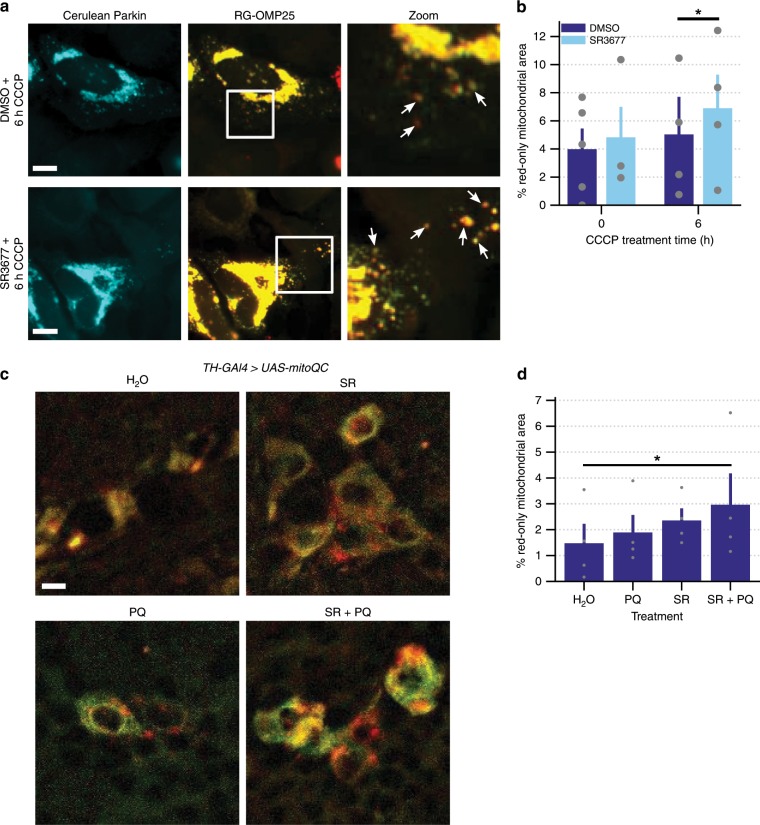
Targeting of mitochondria to lysosomes is increased by SR3677. **a** HeLa cells were co-transfected with Cerulean-Parkin and RG-OMP25. After 24 h, cells were pre-treated with either 0.5 µM SR3677 or DMSO for 2 h prior to induction of mitophagy with 10 µM CCCP treatment, in combination with E-64 and leupeptin. Red-only signal represents mitochondria localized to lysosomes, where GFP signal is quenched. Scale bars, 10 µm. **b** Quantification of the percentage of red-only mitochondrial area divided by the total non-background area (*n* = 4 independent experiments). *P*-values were determined by one-tailed paired Student’s *t*-test. **c** Seven-day-old *TH-GAL4>UAS-mitoQC* male flies were placed into vials containing the indicated treatments. Representative images of the dopaminergic neurons of *TH-GAL4>UAS-mitoQC* flies following feeding on fly food supplemented with H_2_O, 0.5 mM SR3677 (SR) or H_2_O/SR3677 combined with 5 mM paraquat (PQ). Scale bars, 10 µm. **d** Quantification of the percentage of red-only mitochondrial area divided by the total non-background area, averaged across 0.8-µm z-stacks. Data are expressed as mean ± s.e.m (*n* = 4 independent experiments). *P*-values were determined by one-tailed paired Student’s *t*-test, *P < 0.05. Error bars represent s.e.m.

The mito-QC assay measures the targeting of damaged mitochondria to lysosomes. mCherry and GFP are fused to the transmembrane segment of OMP25. Mitochondria localized to lysosomes appear red, rather than yellow due to the acidic quenching of GFP and stability of mCherry in the low pH environment. Consistently, the red dots observed in our assay colocalize with LC3, a lysosomal marker (Supplementary Fig. 23). The targeting of mitochondria to lysosomes is determined by calculating the percentage of the mitochondrial area with red signal only. Pre-treatment of cells with SR3677 increased mitochondrial targeting to lysosomes following induction of mitophagy (Fig. 3a, b). Importantly, SR3677 potentiates but does not induce mitophagy on its own. To confirm that the SR3677-mediated increase to the percentage of red-only mitochondrial area is dependent on autophagy, we tested the effect of SR3677 in cells pre-treated with chloroquine. Chloroquine inhibits the fusion of autophagosomes and lysosomes^37^. We found that pre-treatment with chloroquine abrogates SR3677-mediated enhancement of mitophagy (Supplementary Fig. 23).

The mitoQC assay may also be performed in *Drosophila* to quantify mitophagy specifically in cell types of interest using the GAL4/UAS system. Briefly, we expressed the mitoQC transgene, in dopaminergic neurons using the TH-GAL4 driver^38^. Since CCCP cannot be administered without affecting the viability of the flies, we fed 7-day-old flies the parkinsonian toxin, paraquat. Paraquat has been used to induce mitochondrial dysfunction and to model PD in *Drosophila*. In addition, paraquat is a known inducer of Parkin recruitment^39^. Following 7 days of treatment, fly brains were dissected and imaged. The mitoQC transgene was expressed in dopaminergic neuron clusters in the fly brain. Red dots corresponding to mitochondria localized to lysosomes were evident in all treatments examined, to varying extents. The percentage of red-only mitochondrial signal over the total mitochondrial signal in flies co-treated with SR3677 and a sublethal paraquat dose (1 mM) was greater than flies whose food was supplemented with water (Fig. 3c, d).

### SR3677 enhances mitophagy through inhibition of its canonical target, ROCK2

**Fig. 4:**
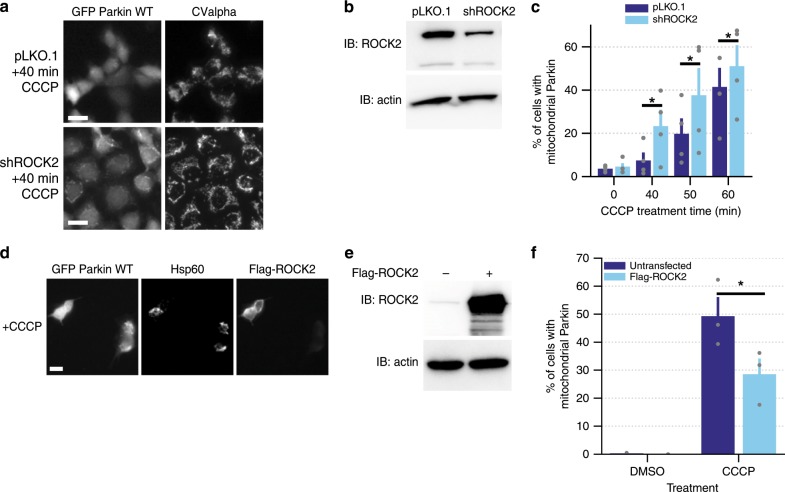
Genetic manipulation of ROCK2 levels mimics effect of SR3677-mediated ROCK2 inhibition. **a** HEK293 GFP Parkin cells stably expressing either pLKO.1 empty control vector or shRNA against ROCK2 (shROCK2) were treated with 10 µM CCCP for 40 min. Immunostaining was performed with anti-CValpha antibody. Scale bars, 10 µm. **b** Cells stably expressing either pLKO.1 empty vector or shRNA against ROCK2 were harvested and proteins were separated by SDS-PAGE. Immunoblotting was performed with anti-ROCK2 and anti-actin antibody. **c** Quantification of the percentage of cells with mitochondrial Parkin following treatment with 10 µM CCCP (*n* = 3 independent experiments). **d** HEK293 GFP Parkin cells were transfected with Flag-ROCK2; 24 h following transfection, cells were treated with 10 µM CCCP. Immunostaining was performed with anti-Flag and anti-Hsp60 antibodies. Scale bars, 10 µm. **e** Untransfected cells and cells transfected with Flag-ROCK2 were harvested and proteins were separated by SDS-PAGE. Immunoblotting was performed using anti-ROCK2 and anti-actin antibodies. **f** Quantification of the percentage of untransfected and Flag-ROCK2 transfected cells with mitochondrial Parkin distribution (*n* = 3 independent experiments). *P*-values were determined by one-tailed paired Student’s *t*-test, *P < 0.05. All graph data are expressed as mean ± s.e.m. All error bars represent s.e.m.

We observed increased Parkin recruitment following administration of six different ROCK inhibitors (Fig. 1a, b). Several of the ROCK inhibitors diverge significantly with respect to their structures, suggesting that these activators likely function by binding ROCK, rather than through a common off-target interaction (Supplementary Fig. 4). Additionally, the specificity of SR3677 has been demonstrated in kinase panel screens^25^. We tested whether genetic manipulation of ROCK2 would phenocopy the effect of SR3677. We generated cell lines stably expressing shRNA against ROCK2 and validated ROCK2 knockdown by western blotting (Fig. 4b, Supplementary Fig. 11). Parkin recruitment increased in ROCK2 knockdown cells (Fig. 4a, c) and decreased in cells transfected with Flag-ROCK2 (Fig. 4d–f). The consistent effect of SR3677 and ROCK2 knockdown on Parkin recruitment and the opposing effect of ROCK2 overexpression demonstrate that ROCK2 negatively regulates Parkin recruitment.

To gain mechanistic insight into how ROCK inhibition upregulates Parkin recruitment to damaged mitochondria, we looked to the pathways that are downstream. ROCK activates PTEN, a negative regulator of Parkin-mediated mitophagy which functions by dephosphorylating mitochondrial ubiquitin, a signal leading to feedforward amplification of Parkin activity^40^. Downstream, PTEN antagonizes the PI3K/Akt pathway^41^. Akt-mediated activation of HK2 and the recruitment of HK2 to mitochondria is required for Parkin recruitment (Supplementary Fig. 12)^12^.

**Fig. 5:**
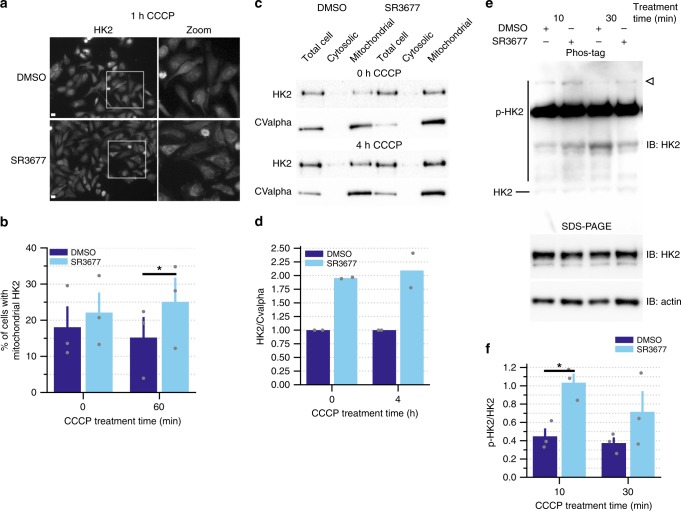
SR3677 activates HK2 and increases its abundance at the mitochondria. **a** HeLa cells were pre-treated with either DMSO or 0.5 µM SR3677 for 2 h prior to addition of 10 µM CCCP for 1 h. Immunostaining was performed against HK2. Scale bars, 10 µm. **b** The proportion of cells with HK2 localized to mitochondria was quantified (*n* = 3 independent experiments). Data are expressed as mean ± s.e.m. **c** SH-SY5Y cells treated with DMSO or 0.5 µM SR3677 were separated into total cell, cytosolic and mitochondrial fractions. Fractions were separated by SDS-PAGE and immunoblotting was performed with anti-HK2 and anti-CValpha antibodies. **d** Densitometry analysis was performed to quantify HK2 levels in each sample, followed by normalization to mitochondrial marker, CValpha. Average HK2/CValpha ratios are plotted (*n* = 2 independent experiments). **e** Serum-starved cells were treated with either DMSO or SR3677 for the indicated time (minutes) prior to harvesting. Lysates were separated by SDS-PAGE and immunoblotting was performed with anti-HK2 and anti-actin antibodies. Samples were also run on Phos-tag gels in parallel and immunoblotting was performed with anti-HK2 antibody. **f** Densitometry analysis was performed on the band indicated by the arrow in **e**. The average intensity of the indicated band normalized to actin loading control is expressed ± s.e.m. (*n* = 3 independent experiments). *P*-values were determined by paired Student’s *t*-test for **b**, **f**, *P < 0.05. All error bars represent s.e.m.

To determine whether HK2 may be involved in the SR3677-mediated effect on Parkin-mediated mitophagy, SH-SY5Y cells were fractionated into total cell, cytosolic and mitochondrial fractions following treatment with either DMSO or SR3677. More HK2 was observed in the mitochondrial fraction following treatment with SR3677 (Fig. 5c, d). Phostag gels were run to assess the phosphorylation status of HK2 following treatment with either DMSO or SR3677. Increased phosphorylation of HK2 was observed following ROCK inhibitor treatment (Fig. 5e, f). Since HK2 is also a Parkin substrate, we used HeLa cells which lack endogenous Parkin to examine the effect of SR3677 treatment on HK2 distribution^42^. SR3677 treatment increases the mitochondrial distribution of HK2 following mitochondrial damage (Fig. 5a, b). Together these results show that SR3677 increases the activity and mitochondrial localization of a positive regulator of Parkin recruitment, HK2.

### The neuroprotective effects of SR3677 in vivo are parkin- and rok-dependent

**Fig. 6:**
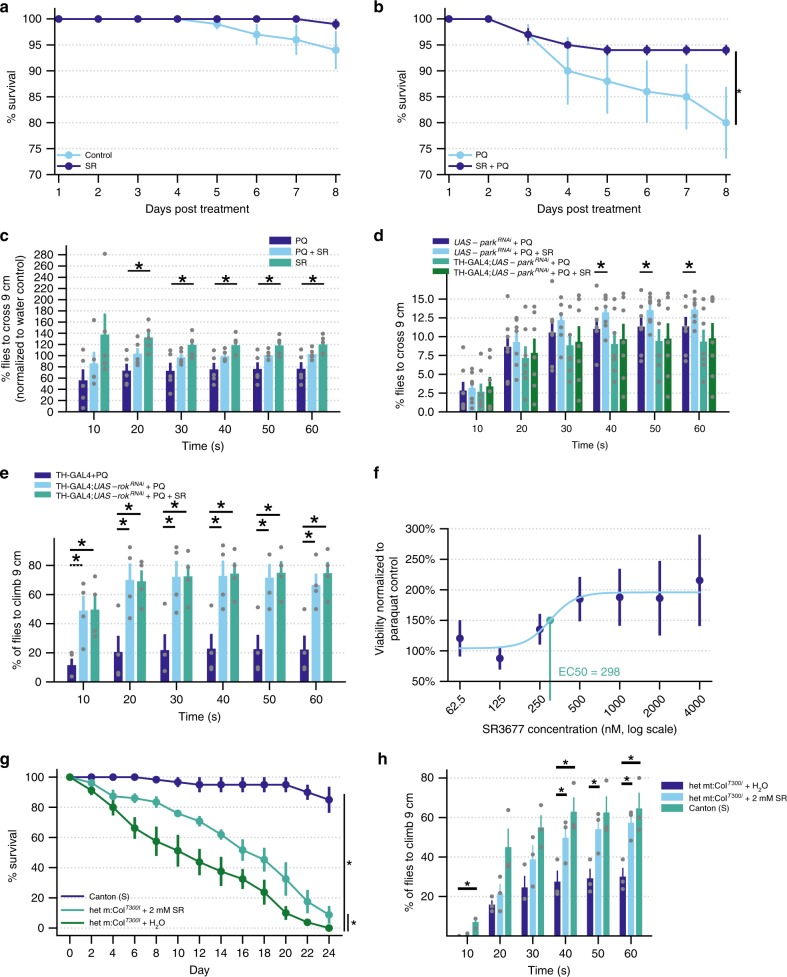
Survival and climbing impairments arising from genetic and chemical mitochondrial dysfunction are improved by SR3677. **a** Seven-day-old male Canton(S) flies were fed water (control), 1 mM SR3677 (SR) (**a**), 10 mM paraquat (PQ) or paraquat in combination with SR3677 (SR + PQ) (**b**). The number of flies alive each day was counted (*n* = 5 independent experiments) and the climbing ability (**c**) of the flies was assessed (*n* = 4 independent experiments). **d** The climbing ability of *UAS-park*^*RNAi*^ and *TH-GAL4*;*UAS-park*^*RNAi*^ (*n* = 4 independent experiments), as well as **e** *TH-GAL4* and *TH-GAL4*;*UAS-rok*^*RNAi*^ flies fed paraquat or paraquat co-administered with SR3677 (*n* = 4 independent experiments). **f** Cell viability, as indicated by ATP levels normalized to protein concentration of differentiated SH-SY5Y cells co-treated with 500 µM paraquat and the indicated concentrations of SR3677 (*n* = 4 independent experiments) normalized to control cells treated with paraquat only. **g** Survival of heteroplasmic *mt:ColI*^*T300I*^ flies fed fly food supplemented with water or 2 mM SR3677 and wild-type Canton(S) flies (*n* = 4 independent experiments). **h** The climbing ability of heteroplasmic *mt:ColI*^*T300I*^ flies fed either water or 2 mM SR3677 for 7 days (*n* = 3 independent experiments). Log-rank tests were performed to determine *P*-values for survival analyses (**a**, **b**, **g**), *P < 0.05. *P*-values were determined by one-tailed paired Student’s *t*-test for all climbing assays (**c**–**e**, **h**), *P < 0.05. Data are expressed as mean ± s.e.m. All error bars represent s.e.m.

To determine whether these findings could be extended in a more relevant cell line, we tested SR3677 in the dopaminergic neuroblastoma SH-SY5Y cell line. Parkin undergoes a similar CCCP-dependent subcellular transition from cytosol to mitochondria in the SH-SY5Y cell line^15^. First, we sought to determine whether the viability of these cells would be improved by SR3677. Crystal violet staining was performed to assess cell viability. Prolonged CCCP treatment (24 h) reduced the amount of staining, indicating the detachment of dead cells from wells. Pre-treatment of cells with SR3677 increased crystal violet staining, as indicated by measuring OD_570_ (Supplementary Fig. 13). Next, SH-SY5Y cells were differentiated according to protocols previously described^43^ and ATP levels were measured to assess viability (Fig. 6f, Supplementary Fig. 14). Treatment with 4 µM SR3677 treatment did not adversely affect cell viability (Supplementary Fig. 15). Varying concentrations of SR3677 were co-administered alongside the parkinsonian toxin paraquat. Notably, SR3677 improved the viability of cells challenged with paraquat in a dose-dependent manner (Fig. 6f).

Next, we sought to determine whether the effects observed in neurons may be recapitulated in a more complex *Drosophila* PD model. Flies with mutations in genes encoding Pink1 and parkin display reduced longevity and reduced locomotor function^44,45^. Flies fed paraquat display similar phenotypes, as a result of mitochondrial dysfunction^46,47^. We used this neurotoxin model to test the effect of SR3677 on PD-related phenotypes. We aligned the amino acid sequences of *Drosophila* Rho-associated kinase (rok) to ROCK2, the human isoform with which it shares the greatest sequence similarity. The amino acids predicted to be essential for binding of SR3677 to human ROCK2 (Met-172, Glu-170, Lys-121, Asp-176) are conserved in the *Drosophila* rok sequence^25^.

Seven-day-old Canton(S) male flies were fed their standard diet supplemented with paraquat and SR3677 (Supplementary Fig. 16). The survival of flies was reduced following administration of paraquat, as reported by others^47–49^. Co-administration of SR3677 improved the longevity of flies challenged with paraquat (Fig. 6b). Feeding SR3677 alone does not affect the survival of flies (Fig. 6a). Paraquat administration impairs the climbing ability of flies (Fig. 6c). Co-treatment with SR3677 restores climbing ability in paraquat-treated flies. To verify that flies were consuming paraquat and SR3677, we supplemented the treatments with blue food dye and observed blue coloring in the abdomens of the flies (Supplementary Fig. 17).

We also assessed whether SR3677 may protect against paraquat-induced phenotypes by interfering with paraquat’s activity. Paraquat gives rise to mitochondrial superoxide species that can be detected using mitoSOX, a chemical probe that is targeted to mitochondria, where it fluoresces when it encounters superoxides. The fluorescence intensity of mitoSOX-stained Schneider’s S2-R^+^ cells, a cell line derived from dissociated fly embryos with a flat morphology amenable to imaging, was assessed following treatments with paraquat or paraquat and SR3677. Treatments with both paraquat and luperox, a tert-butyl hydroperoxide increase the fluorescence intensity of mitoSOX. Co-treatment with SR3677 does not impede paraquat-mediated superoxide generation (Supplementary Fig. 18).

Previous studies have described the neuroprotective effect of ROCK inhibition in other PD models^19,20,50–52^. General autophagy enhancement or other downstream Akt substrates were attributed with the neuroprotective effects of ROCK inhibition^19,20,50^. To test whether the Parkin-mediated mitophagy pathway may be required for the neuroprotective effects observed following ROCK inhibitor treatment, we crossed tyrosine hydroxylase *(TH)-GAL4* flies with transgenic *UAS-park*^*RNAi*^ flies to specifically knockdown Parkin in the dopaminergic neurons of the fly. These flies and the parental *UAS-park*^*RNAi*^ fly line were fed vehicle control (water) or SR3677 along with paraquat. The climbing ability of the *UAS-park*^*RNAi*^ flies was improved when SR3677 was co-administered alongside paraquat, as demonstrated in Canton(S) flies. SR3677 co-administration produced no improvement to paraquat-induced climbing defects in *TH-GAL4; UAS-park*^*RNAi*^ flies (Fig. 6d, Supplementary Fig. 19). We also found that *TH-GAL4; UAS-rok*^*RNAi*^ flies fed paraquat displayed improved climbing ability compared to *UAS-rok*^*RNAi*^ flies fed paraquat, demonstrating that RNAi mediated knockdown of SR3677’s canonical target phenocopies its effect on climbing deficits. No further improvement to climbing ability was observed in *TH-GAL4; UAS-rok*^*RNAi*^ flies fed SR3677 alongside paraquat (Fig. 6e) indicating the requirement for rok.

Finally, we tested SR3677 in an alternate, genetic model of mitochondrial dysfunction. Specifically, we used flies with a temperature-sensitive, de-stabilizing mutation in cytochrome c oxidase subunit I (*mt:Col*^*T300I*^). This mutation causes depolarization of the mitochondrial membrane potential and increased mitochondrial reactive oxygen species^53^, manifesting in systemic consequences such as impaired climbing and survival. Flies with this mutation in 100% of their mitochondrial genomes, or homoplasmic flies, only survive 4 days post-eclosion. In order to extend our therapeutic window, we tested SR3677 in heteroplasmic flies which contain this temperature-sensitive mutation in ~90% of their mtDNA instead^54^. These flies retain the same phenotypes as homoplasmic flies, including reduced survival and climbing ability although to a less severe extent (Fig. 6g, h).

Immediately following eclosion, we transferred male heteroplasmic *mt:Col*^*T300I*^ flies to vials containing either water or 2 mM SR3677. Newly eclosed Canton(S) male flies were placed into fresh vials and referenced as a control and flies were raised at non-permissive 29 °C for these experiments. Survival was improved by 2 mM supplementation of SR3677 into fly food. Climbing ability was also improved by 2 mM SR3677 addition. In agreement with the effect of SR3677 co-administration on paraquat-mediated survival and climbing deficits (Fig. 6a–c), this model replicates the improvement to phenotypes arising from mitochondrial dysfunction.

## Discussion

In our study, we find that ROCK negatively regulates Parkin-mediated mitophagy and by inhibiting ROCK using small molecules, this brake on Parkin-mediated mitophagy is released. Numerous downstream substrates are activated by ROCK, including PTEN, a negative regulator of Akt and recently identified negative regulator of Parkin^40,41^. McCoy et al. have demonstrated the requirement for Akt-mediated activation of HK2 for the recruitment of Parkin to damaged mitochondria. Consistently, over-expression of HK2 increases Parkin recruitment. Following treatment with ROCK inhibitor, we observe increased HK2 activation and mitochondrial subcellular distribution. Through this mechanism, it is possible to upregulate Parkin-mediated mitophagy without reducing the viability of cells. This contrasts with most activators of Parkin-mediated mitophagy, which function through mechanisms of action involving mitochondrial damage and/or apoptosis^17^.

Recent studies have found that F-actin cages encapsulate depolarized mitochondria, leading to their degradation by mitophagy^55^. Since ROCK2 phosphorylates cofilin, resulting in the stabilization of the actin cytoskeleton^56^, we cannot exclude the possibility that ROCK inhibitors may upregulate mitophagy by destabilizing the actin cytoskeleton, which may facilitate the formation of F-actin cages around depolarized mitochondria. Since F-actin cage assembly is downstream of Parkin recruitment to mitochondria and Parkin is required for the rescue of paraquat-induced locomotor impairment, we speculate that the effect can mostly be attributed to the increased levels of mitochondrial HK2. After validating the upregulation of Parkin-mediated mitophagy at several steps including the recruitment of Parkin to damaged mitochondria, the clearance of outer mitochondrial membrane substrates and the targeting of mitochondria to lysosomes in cells and directly in dopaminergic neurons, we tested whether this manipulation could improve phenotypes associated with PD. Differentiated SH-SY5Y cells and *Drosophila* treated with SR3677 demonstrated improved survival when challenged with the PD-causing toxin paraquat. Notably, the locomotor ability of paraquat-fed flies was improved by SR3677 and by RNAi-mediated knockdown of rok in the dopaminergic neurons.

In an alternate, genetic model of mitochondrial dysfunction caused by a destabilizing mutation which abolishes cytochrome c oxidase activity, we also found that SR3677 improves climbing and survival deficits. This can likely be attributed to the SR3677-mediated enhancement of mitophagy, which we observed in vivo using the mitoQC assay (Fig. 6c, d). To perform these experiments, we used a heteroplasmic model of mtDNA mutation to extend our therapeutic window. Interestingly, in experiments performed in cells, Parkin has been found to decrease levels of heteroplasmy^57^. Heteroplasmic mtDNA deletions are increased in the substantia nigra of PD patients and the levels of mtDNA containing these deletions increases with age in both individuals with PD and in control subjects^58^. It remains to be tested whether degradation of mutant mtDNA-containing fragments is responsible for the SR3677-mediated improvements to survival and climbing ability in the het *mt:ColI*^*T300I*^ flies.

Previous studies have observed that several ROCK inhibitors reduce tau levels in neurons through autophagy enhancement^19,20^. This translates into suppression of a rough eye phenotype caused by tauopathy in *Drosophila*, indicating efficacy in vivo. General autophagy upregulation was attributed for ROCK inhibitor-mediated neuroprotection in these studies. Interestingly, recent work demonstrates that mitophagy induction inhibits tau pathogenesis and improves phenotypes arising from tauopathy in *Caenorhabditis elegans* and mice^59^. Considering these studies, in addition to our own, mitophagy induction should further be investigated as a central mechanism governing the neuroprotective effects of ROCK inhibitors.

While previous studies have similarly documented the neuroprotective effect of ROCK inhibition in PD models, we established that in dopaminergic neurons, these effects were Parkin-dependent. To our knowledge, this is the first study to connect the neuroprotective effect of ROCK inhibition to Parkin-mediated mitophagy upregulation. By gaining insight into the mechanism of action of these promising small molecules, it will be possible to apply them most effectively, including to rationally design combinatorial therapies.

Interestingly, in Japan, a ROCK inhibitor called Fasudil and its derivative have been used for the treatment of vasospasm following subarachnoid hemorrhage. Since no adverse effects have been reported and this compound has demonstrated efficacy in vivo, this drug may be an ideal candidate for repurposing. Upon re-testing Fasudil, we observed increased mitochondrial Parkin distribution following induction of mitochondrial damage (Supplementary Fig. 21). Fasudil improves the survival of paraquat-treated flies and improves climbing deficits, although not to a statistically significant extent at the same dose at which we tested SR3677 (1 mM). Dose escalation may increase its efficacy in this severe model of mitochondrial dysfunction.

The advantages of Fasudil, a drug with a known safety profile should be balanced by the advantages of other ROCK inhibitors which are ROCK2-specific. As mentioned previously, ROCK2 is enriched in neurons^22^, so pharmacologically targeting this isoform would lessen side effects which may arise from systemic ROCK1 and ROCK2 inhibition. While the poor bioavailability of SR3677 should be noted, the analog SR3850 that was not represented in our screen which demonstrates superior pharmacokinetic properties can be tested^60^.

Future attempts to increase the pool of Parkin localized to damaged mitochondria may benefit from two strategies: (1) to activate HK2 directly or (2) to inhibit negative regulators of the Akt-HK2 axis. The first strategy may employ a similar method to the structure-guided design of neo-substrates, as has been used to successfully identify PINK1 activators^5^. For the second strategy, inhibitors of negative regulators of Akt, such as PTEN, may be screened for their ability to potentiate Parkin. The most potent and selective inhibitor of PTEN identified to date is SF1670 (ref. ^61^). While our screening efforts included ~3000 molecules, PTEN inhibitors were not represented in our dataset. These compounds can be tested alone and in combination with the ROCK inhibitors identified in our study, given that they may have an additive effect by enhancing a common pathway. Increasing the capacity of dopaminergic neurons to eliminate damaged mitochondria may lead to the development of much-needed disease-modifying therapeutics for the treatment of PD and other diseases characterized by mitochondrial dysfunction.

## Methods

### Stable cell line development

GFP Parkin plasmids, a gift from Dr. Wolfdieter Springer, were transfected into HEK293 cells using Lipofectamine2000 (Invitrogen, 11668027) according to manufacturer’s instructions. Cells stably expressing GFP Parkin were selected using 800 µg/mL geneticin (Gibco, 11811031). FACS sorting was performed to select for cell populations expressing GFP at similar levels.

HEK293 GFP Parkin cells were transfected with shRNA targeting ROCK2 or with pLKO.1 control vector (Sigma, SHC001). Puromycin (Biobasic, PJ593) selection was performed to select for cells stably expressing this construct. ROCK2 KO cell lines were generated using CRISPR/Cas9 gene editing. Briefly, we designed gRNA targeting sites within the first exon of ROCK2 using http://crispr.mit.edu/. Two oligonucleotides (5′ CACCGATGAGCCGGCCCCCGCCGAC-3′ and 5′-AAACGTCGGCGGGGGCCGGCTCATC-3′) containing the target sequences were annealed and cloned into the PX458 vector (Addgene plasmid #48138). Following transfection of this construct or the parental PX458 vector into HEK293 cells, single GFP-positive cells were sorted into 96-well plates for colony isolation.

### Small-molecule screening

A total of 10 µL of 0.1 mg/mL Poly-d-Lys solution was added to 384-well plates (Corning #3712) for 5 min, followed by a PBS wash. Plates were dried for at least 2 h; 50 µL of 600000 HEK293 GFP cells/mL of DMEM was dispensed into each well. After 24 h incubation to allow the cells to adhere, 200 nL of small molecules (or DMSO in columns 1, 2, 23 and 24) were pinned. Following a 16-h incubation, 200 nL of 5 mM CCCP was added to all wells except columns 1 and 2, into which 200 nL of DMSO was added. Following 2-h incubation, cells were fixed with 4% paraformaldehyde (PFA) and stained with 50 µL of 1 µg/mL DAPI solution for 15 min. After the final PBS wash, plates were ready for high content microscopy.

Images were acquired on IN Cell Analyzer 6000 (GE Healthcare), equipped with sCMOS camera (2048 × 2048), and 20×/0.45 NA Plan Fluor objective (Nikon) in open aperture mode using 1 × 1 binning. Image analysis was performed using Columbus Image Analysis System (PerkinElmer). Nuclei were initially detected in DAPI channel, followed by whole-cell segmentation in GFP channel. Using PhenoLOGIC machine learning plug-in, cells were categorized into two sub-populations: cells with even GFP-Parkin distribution and cells with GFP-Parkin localized to mitochondria. The percentage of cells with mitochondrial GFP-Parkin was determined per well and averaged across two independent trials.

### Hit selection

Compounds with average activity values greater than 80% were considered candidate activators, while those with values below 50% were considered inhibitors. The activators and inhibitors were grouped according to their protein targets. Based on both the number of activators belonging to each chemical family and on their average activity score, the ROCK inhibitor series was selected. Specifically, we followed up on three ROCK inhibitors with a high degree of structural similarity (Supplementary Fig. 4).

### Chemical similarity determination

SMILEs for all activator compounds were obtained using the Python library PubchemPy (https://pubchempy.readthedocs.io), which provides programmatic access to the Pubchem compound database (https://pubchem.ncbi.nlm.nih.gov/). SMILEs were converted into a Morgan fingerprints using the Python Library RDKit^45^. Principle component analysis was conducted to reduce the dimensionality of the data, using the Python library scikit-learn (http://scikit-learn.org/stable/). This allows for evaluation of chemical similarity based on the proximity of their corresponding data points in 2D space.

### Protein target similarity determination

Protein target information was provided by the chemical library supplier or acquired from Drugbank database (https://www.drugbank.ca/). Activators were grouped based on common targets and average activity values (% of cells with mitochondrial Parkin) were determined for each family of compounds with a common target. Screening leads were prioritized for validation according to the average activity values and the number of molecules belonging to each family of activators (Fig. 1b).

### Chemicals

The following chemicals were used in our study: CCCP (Sigma, C2759), SR3677 (Tocris, 3667/10), Y27632 (Millipore, 688000), Y39983 (MedChemExpress, HY-10069), paraquat (Sigma, 36541), E-64 (BioShop, EEL640.1), leupeptin (BioShop, Leu001.50), geneticin (Gibco, 11811023), puromycin (BioShop, PUR333.10), retinoic acid (Calbiochem, 554720), brain-derived neurotrophic factor (Gibco, PHC7074) and chloroquine (Bioshop, CHL919).

### Constructs and shRNA

Cerulean-Parkin, RG-OMP25 were a gift from Dr. Peter Kim and have previously been described^36,62^. R777-E285 Hs.ROCK2 was a gift from Dr. Dominic Esposito (Addgene plasmid #70569). The Gateway recombination system (Life Technologies) was used to insert ROCK2 into pDEST-pcDNA5-FLAG^63^.

ShRNA against ROCK2 (5′-CCGGCCTTGATGTCTGTCTATTATTCTCGAGAATAATAGACAGACATCAAGGTTTTTTG-3′) was purchased (Sigma, TRCN0000184636). Empty control pLKO.1 vector was a gift from Dr. Alex Palazzo.

The following oligonucleotides were phosphorylated with T4 polynucleotide kinase (NEB, M0201S) and annealed in a thermocycler: 5′-CACCGATGAGCCGGCCCCCGCCGAC-3′and 5′-AAACGTCGGCGGGGGCCGGCTCATC-3′ and inserted into the PX458 vector (Addgene plasmid# 48138).

The following primers were used to amplify ROCK2 from R777-E285 Hs.ROCK2 plasmid (addgene plasmid# 70569):

5′-ATAAGAGGATCCGCCGCCACCATGAGCCGGCCCCCGCCGACGGGGAAAAT-3′ and 5′-ATAAGACTCGAGTTAGCTAGGTTTGTTTGGGGCAAGCTGTCGACTTGG-3′. ROCK2 was then inserted into pDEST-pcDNA5-BirA-FLAG N-term. The following primers were used to remove BirA: 5′-CATAGAAGACACCGGGACC-3′ and 5′-GCGCCTAGTTTATCGTCATCG-3′.

### Antibodies

The following primary antibodies were used in our study: mouse anti-CValpha (Abcam, 14748; 1:5000), rabbit anti-Hsp60 (Abcam, 46798; 1:500), rabbit anti-ROCK2 (Abcam, 125025; 1:5000), mouse anti-actin (Abcam, 8226; 1:1000), mouse anti-HK2 (Abcam, 3740910; 1:1000), mouse anti-Mfn2 (Abcam, 56889; 1:1000), rabbit anti-TOM20 (Santa Cruz, 191883; 1:1000), mouse anti-VDAC1 (Abcam, 14734; 1:1000), mouse anti-TOM70 (Santa Cruz, 390545; 1:1000), mouse anti-UQCRC2 (Abcam, 14745, 1:1000), rabbit anti-COXIV (Novus, NB110-39115; 1:500) and mouse anti-Flag (Sigma, F1804; 1:1000). Rabbit and mouse horseradish peroxidase-conjugated secondary antibodies (Jackson Immunoresearch; 1:5000) were used for secondary incubation.

The following primary antibodies were used in our study for immunofluorescence: mousen anti-CValpha (Abcam, 14748; 1:500), rabbit anti-Hsp60 (Abcam, 46798; 1:500), rabbit anti-LC3B (Cell Signaling, 2775) and mouse anti-Flag (Sigma, F1804; 1:1000). All antibodies were diluted in 1% goat serum.

### Cell culture

Cells were cultured in Dulbecco’s modified Eagle’s Medium (DMEM) supplemented with 10% fetal bovine serum (Sigma, F1051) at 37 °C in humidified air containing 5% CO_2_. Cells were tested for mycoplasma contamination (Lonza, LT07).

### Immunoblotting

Lysates were harvested using lysis buffer (0.1 M Tris HCl, 0.01% SDS, pH 9) with 1× protease inhibitor cocktail (BioShop, PIC002.1). Lysates were then heated at 95 °C for 20 min with vortexing performed at 5-min intervals. The BCA assay (Pierce, 23227) was performed to determine protein concentration, to standardize protein loading across samples to be compared; 10% SDS-PAGE gels were run for Mfn2 and ROCK2 immunoblotting and 12% gels for all other experiments. Transfer of proteins onto PVDF membrane (Immobilon, IPVH00010) was performed at 110 V for 80 min (with ice pack to cool apparatus) or at 36 V for 8 h at 4 °C. Membranes were blocked with 5% skim milk (BioShop, SKI400.500) in TBST (1× TBS, 0.1% Tween-20, BioShop, 1M23298) for 30 min prior to incubation in primary antibodies at a dilution of 1:1000 for all antibodies except anti-ROCK2 and anti-LC3, which were both used at a dilution of 1:5000. Visualization of proteins was performed using ECL (BioRad, 11705062).

Phostag western blotting was carried out using the same protocol except SDS-PAGE gels include 5 µM Phos-tag^TM^ Acrylamide (Wako, 300–93523) and following electrophoresis, the gel was washed three times with transfer buffer containing 10 mM EDTA for 10-min intervals and one final wash with transfer buffer without EDTA prior to protein transfer.

Densitometry was performed by normalizing bands corresponding to the protein of interest to a loading controls such as actin or Ponceau staining using ImageLab 6.0 software (BioRad).

### Immunofluorescence

Cells on coverslips were washed with PBS, incubated in 4% PFA diluted in PBS for 15 min, washed again with PBS prior to incubation in 0.1% Triton X-100 for 15 min. After a final PBS wash, coverslips were then incubated in 10% goat serum (Gibco, 16210072) for 30 min at room temperature. Next, coverslips were incubated in primary antibodies diluted 1:500 in PBS at 4 °C overnight. Following a PBS wash, coverslips are incubated with Alexa Fluor secondary antibodies (Life Technologies, A-11001, A-11004 and A-11011) at a concentration of 1:500 for 2 h, PBS washed and mounted onto slides using Fluoromount-G (Invitrogen, 00495802). Images were acquired.

### Mito-QC assay

HeLa cells were seeded into 6-well plates containing glass coverslips and allowed to adhere overnight. Cells were co-transfected with RG-OMP25 and Cerulean-Parkin plasmids 24 h prior to treatments. E-64 and leupeptin were added along with either DMSO or 10 µM CCCP for 6 h prior followed by a PBS wash, fixation with 4% PFA for 15 min and a final PBS wash prior to mounting onto slides with Fluoromount. Cells were treated with 50 µM chloroquine for 16 h prior to addition of E64, leupeptin, SR3677 and CCCP in the indicated treatment group.

Quantification of the RG assay was performed using Fiji software. A region of interest (ROI) encompassing all signal was drawn around each cell and a red/green fluorescence intensity ratio was determined for each pixel within the ROI. Pixels were considered red-only if the red/green fluorescence ratio was ≥2. The area of red-only pixels over the total non-background area was determined for each cell and averaged across three independent trials in which at least 20 cells were quantified per treatment group.

For the in vivo mitoQC assays, five 7-day-old male TH-GAL4>UAS-mitoQC were placed into vials containing water (control), sub-lethal 1 mM paraquat, 1 mM SR3677 alone and in combination with paraquat for 7 days. Whole flies were then fixed overnight in 1% PFA and 0.1% Tx-100. Fly brains were then dissected and fixed with 4% PFA for 20 min. Three 10-min PBS washes were performed in between each step. Fly brains were mounted in VectaShield mounting medium for imaging using the Zeiss LSM700 confocal fluorescence microscope with 40 × 1.4 NA Oil Plan-APOCRAMAT objective and the appropriate lasers and filter; 0.8 µm z-stacks were acquired to capture dopaminergic neurons expressing the mitoQC transgene. Laser settings were kept constant within each trial. At least two fly brains were imaged for each treatment across four independent trials.

Quantification of the RG assay was performed using Cell Profiler to segment cells, subtract background and to quantify the mCherry/GFP ratio of all non-background pixels. Pixels with mCherry/GFP ratio above 1.5 were considered red-only. The area of red-only pixels over the total non-background area was determined for each z-stack and the values were averaged for each ROI. At least ten independent regions of interest per treatment were imaged across four independent trials.

### Mitochondrial clearance assay

HeLa cells stably expressing GFP Parkin and mito-DsRed were seeded into 12-well plates containing glass coverslips and allowed to adhere overnight. Cells were then treated with DMSO or 0.5 µM SR3677 for 2 h prior to addition of 10 µM CCCP for 24 h. Cells were then washed with PBS, fixed with 4% PFA for 15 min and washed with PBS again prior to the mounting of coverslips onto glass slides. Mitochondrial clearance was quantified as the percentage of cells in each treatment which retain mito-DsRed signal. Three independent trials were performed with at least 50 cells quantified per trial.

### Parkin recruitment assay

HEK293 GFP Parkin cells were seeded into 12-well plates containing coverslips. Cells were treated with small molecules for 2 h prior to mitophagy induction with 10 µM CCCP, unless indicated otherwise. Following fixation with 4% PFA, immunostaining was carried out using anti-CValpha primary antibody and anti-mouse Alex Fluor 568.

To measure the percentage of cells with Parkin localized to mitochondria, we employed a two-step process: (1) whole-cell segmentation based on GFP Parkin signal using CellProfiler software and (2) classification into Parkin distribution subpopulations (cytosolic or mitochondrial) using CellProfiler Analyst. The percentage of cells with mitochondrial Parkin was determined in each treatment. Images were taken at 20× magnification for this experiment and at least 200 cells in each treatment group.

### Mitochondrial isolation

Two wells of a 6-well plate were fractionated into total, cytosolic and mitochondrial fractions using the Mitochondrial Isolation Kit for Cultured Cells (Abcam, 110170). Samples were separated via SDS-PAGE. CValpha was used as a mitochondrial marker.

### Cell viability determination

SH-SY5Y cells were differentiated through sequential treatment with retinoid acid following by brain-derived neurotrophic factor (Cell Signaling, 3897S)^43^. Cells were then seeded into white-bottom 96-well plates (CellStar, 655083) and allowed to adhere overnight. Varying doses of SR3677 were combined with 500 µM paraquat and administered for 24 h. Controls treated with 0.5 µM SR3677, equivalent volume DMSO and paraquat alone were also included. Following 24-h incubation, cell medium was aspirated and TNE lysis buffer (50 mM Tris-HCl, 100 mM NaCl, 0.1 mM EDTA) containing protease inhibitor cocktail was added to each well. Half of the lysate volume was removed and aliquoted into a separate 96-well plate for protein concentration measurement using the Pierce BCA (Thermo Fisher, 23225) assay kit. ATP levels were measured in the opaque plate using the ATP determination (Thermo Fisher, A22066) assay kit and protein concentrations. Three technical replicates were averaged and the resulting ATP levels were normalized to protein concentration and then by the ATP/[protein] ratio for cells treated with paraquat alone in a control well on each plate.

### Drosophila stocks

All stocks were maintained at 25 °C and at 70% relative humidity in 12-h light/dark cycles and were fed standard yeast-molasses-agar medium. The following fly lines were obtained from Bloomington Drosophila Stock Center: *UAS-park*^*RNAi*^ (Bloomington stock #: 31259), *UAS-rok*^*RNAi*^ (Bloomington stock #: 34324), *TH-GAL4* (Bloomington stock #: 8848), *tubulin-GAL4* (Bloomington stock #5138). *Canton (S)* and heteroplasmic *mtColI*^*T300I*^ flies were generously provided by Dr. Thomas Hurd (University of Toronto).

### Drosophila longevity and climbing assays

Male Canton(S) *Drosophila* were aged 7 days prior to feeding with standard fly food supplemented with water, 10 mM paraquat, 1 mM SR3677, or with combinations of these chemicals. Flies were placed into new vials containing fresh food supplemented with the treatments previously administered and climbing assays were performed 4 days later. Flies were placed into plastic cylinder vials and tapped down, so all flies fell to the bottom. The number of flies to climb beyond 12.5 cm was determined at 10-s intervals and climbing ability values are represented as the percentage of total flies in each treatment group. Four technical replicates were performed on each treatment group and the average was determined.

Heteroplasmic mt*Col*^T300I^ flies were raised at the non-permissive temperature of 29 °C. Twenty newly eclosed flies were placed into vials containing standard fly food supplemented with water or SR3677. Flies were placed into fresh vials every 2 days, at which point survival was assessed. Flies were treated the same way for climbing assays, which were performed after 6 days of treatment. The percentage of flies to climb beyond 9 cm at 10-s intervals was determined to assess climbing ability. Four technical replicates were performed on each treatment group and the average was determined.

### Statistical analysis

Paired Student’s *t* tests were used for all analyses, unless otherwise specified. Logistic regression was performed to construct dose response curves, which was used to determine EC_50_ values. Survival analyses were performed using the log-rank test to analyze whether a difference in the survival rates between treatments was significant. At least three independent biological replicates were performed for all experiments for which statistical analysis was performed. All figure legend sample sizes (*n*) refer to the number of independent experiments performed. *P*-values < 0.05 were considered significant.

### Reporting summary

Further information on research design is available in the Nature Research Reporting Summary linked to this article.

## Supplementary information

Supplementary Information

Description of Additional Supplementary Files

Supplementary Data 1

Reporting Summary

## Data Availability

The source data for all graphs are provided.

## Source Data

Source Data

## Code availability

Code is available upon request.
