# Supplementary material for: ROCK inhibitors upregulate the neuroprotective Parkin-mediated mitophagy pathway
Source: Nat Commun. 2020 Jan 3;11:88. doi: 10.1038/s41467-019-13781-3 (PMC6941965; doi:10.1038/s41467-019-13781-3)
Supplement: Supplementary file 1 — Supplementary Information [file 41467_2019_13781_MOESM1_ESM.pdf]

ROCK inhibitors upregulate the neuroprotective Parkin-mediated mitophagy pathway

Natalia Moskal[1], Victoria Riccio[1], Mikhail Bashkurov[2], Rediet Taddese[1], Alessandro Datti[2, 3],

Peter N. Lewis[1], G. Angus McQuibban[1*]

Supplementary Data

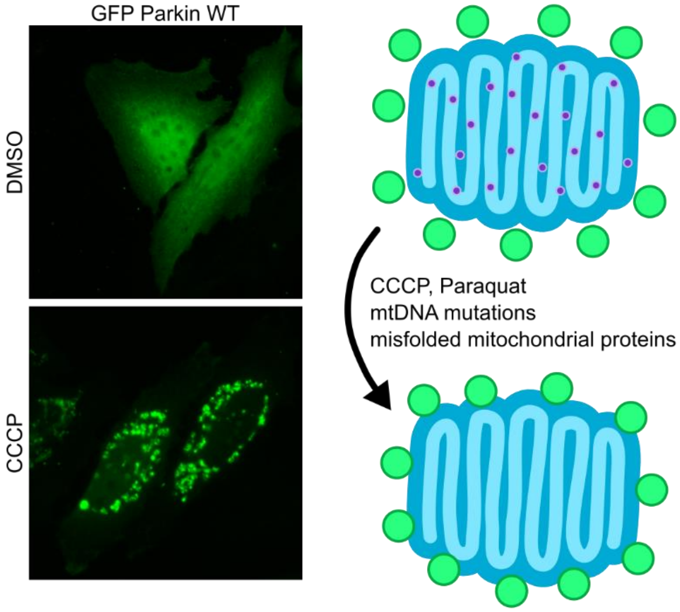

Supplementary Figure 1: HeLa cells expressing GFP-Parkin WT were treated with either DMSO or 20 µM CCCP. Parkin undergoes a subcellular transition from the cytosol to the mitochondria following CCCP treatment. In addition to chemicals such as CCCP, which induce mitochondrial damage, mitochondrial DNA mutations and accumulation of misfolded mitochondrial proteins also cause Parkin to translocate to mitochondria.

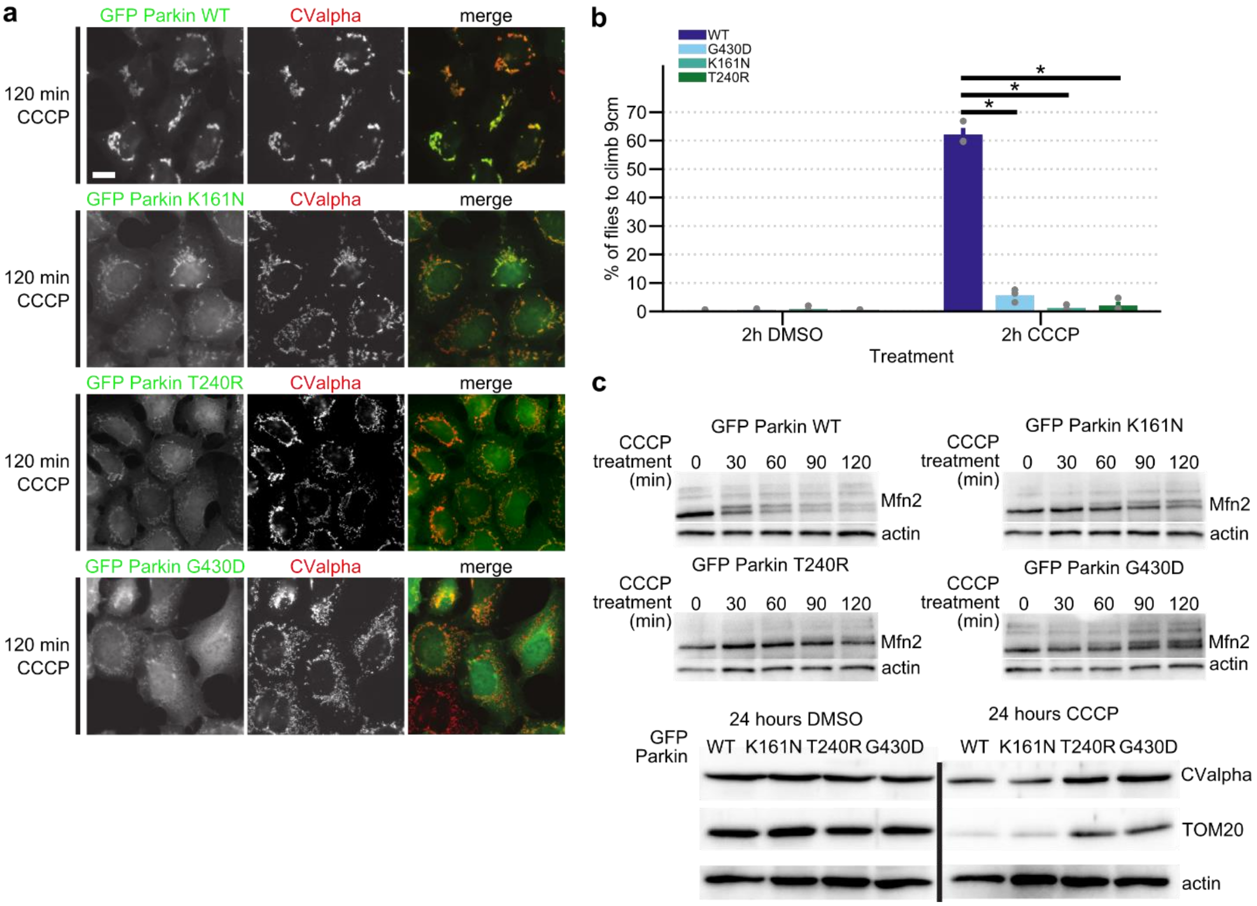

Supplementary Figure 2: PD-linked Parkin mutants fail to localize to damage mitochondria, leading to the impaired degradation of mitochondrial proteins. (a) GFP Parkin WT, K161N, T240R and G430D expressing HEK293 cells were treated with 20 µM CCCP to induce mitophagy. Immunostaining was performed to visualize the mitochondrial marker CValpha. Scale bars, 10 µm. (b) Quantification of the percentage of cells with Parkin localized to mitochondria following 2 hour treatment with either DMSO or CCCP (n=3 independent experiments). P-values were determined by one-tailed paired student's t-test, *P-value<0.05. Data is expressed as mean± s.e.m. Error bars represent s.e.m. (c) Cells were treated with CCCP for the indicated time intervals. Cell lysates were then separated by SDS-PAGE and immunoblotting was performed against the mitochondrial proteins Mfn2, CValpha and TOM20. Actin was used as a loading control. Representative blots displayed, n=3 independent experiments performed with similar results.

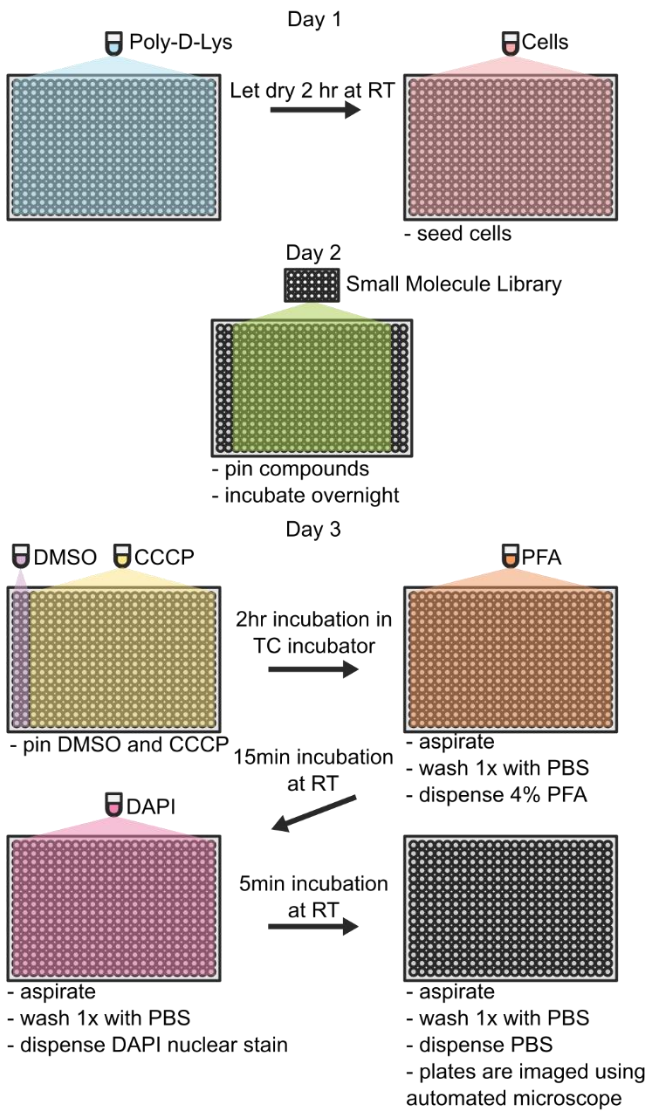

Supplementary Figure 3: Screening workflow and image analysis. On day 1, 4,000 HEK293 GFP Parkin cells were seeded onto Poly-D-Lys coated 384-well plates. On Day 2, cell media was exchanged for new media containing 4 µM of compound. On Day 3, 20 µM CCCP was administered, cells were fixed with 4% paraformaldehyde followed by DAPI staining. PBS washes were performed between each step following administration of CCCP. High content microscopy was performed to visualize the cells within each well. Cells in each image could be subdivided into two phenotypic classes: cells with mitochondrial Parkin distribution and cells with cytosolic Parkin distribution. Each well was scored for the percentage of cells with mitochondrial Parkin distribution.

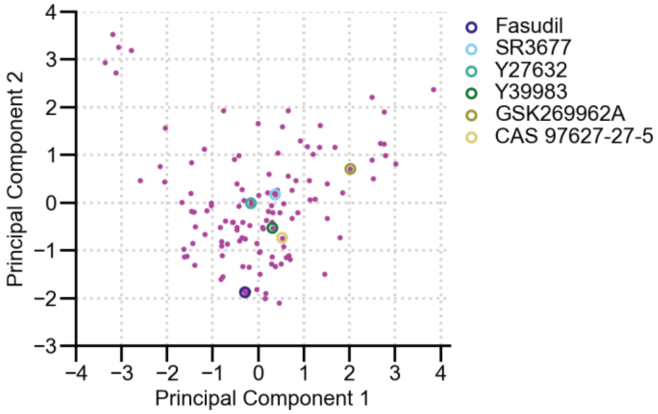

Supplementary Figure 4: Principal Component Analysis of Morgan fingerprints of activator compounds. The proximity of two data points is indicative of the degree of structural similarity between two compounds. ROCK inhibitors Fasudil (navy), SR3677 (light blue), Y27632 (light green), Y39983 (green), GSK269962A (olive) and CAS 97627-27-5 (yellow) are indicated amongst the activators (magenta dots).

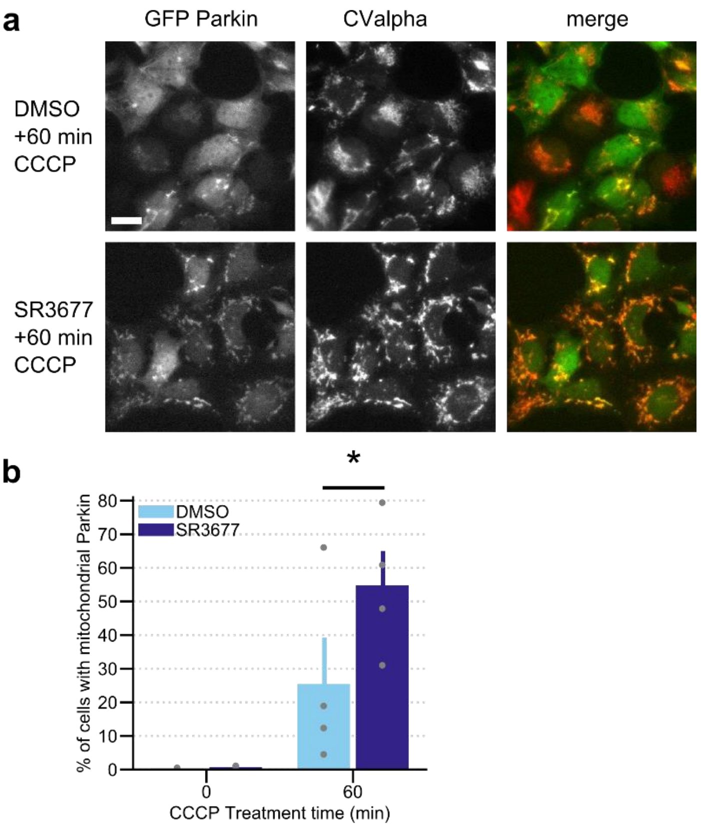

Supplementary Figure 5: (a) GFP Parkin WT cells pre-treated with either DMSO or 0.5 µM SR3677 were treated with 10 µM CCCP for 1 hour. Scale bars, 10µm. (b) Quantification of the percentage of cells with mitochondrial Parkin (n=4 independent experiments). P-values were determined by one-tailed paired student's t-test, **P-value*<0.05. Data is expressed as mean ± s.e.m. Error bars represent s.e.m.

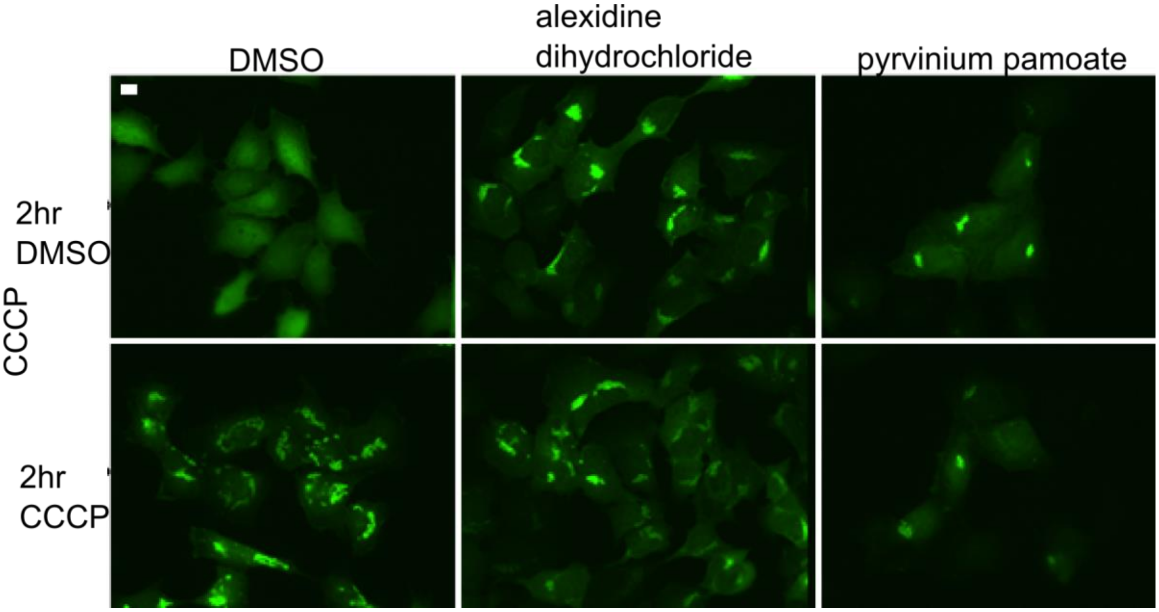

Supplementary Figure 6: Parkin distribution in HEK293 GFP-Parkin cells treated with DMSO, 4 µM alexidine dihydrochloride or 4 µM pyrvinium pamoate for 16 hours prior to addition of DMSO or CCCP. Representative images displayed, n=3 independent experiments performed with similar results. Scale bars, 10µm.

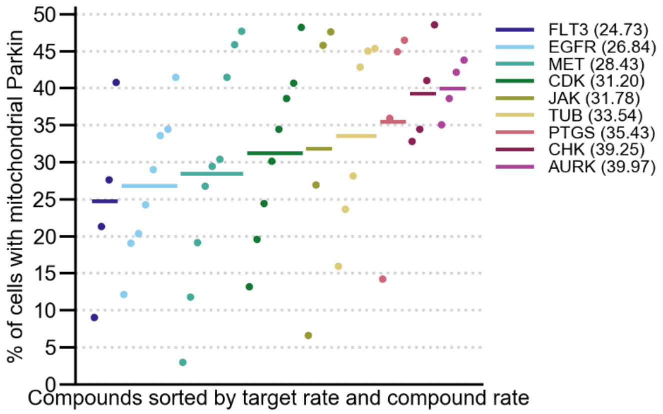

Supplementary Figure 7: Parkin recruitment inhibitors identified in high throughput screen were grouped according to their canonical target. The average activity value (% of cells with mitochondrial Parkin) for each compound is represented by each dot and the average for each group is represented by each dash. The small molecule groups with common canonical targets are displayed in ascending order, based on the average activity value of all compounds within the group.

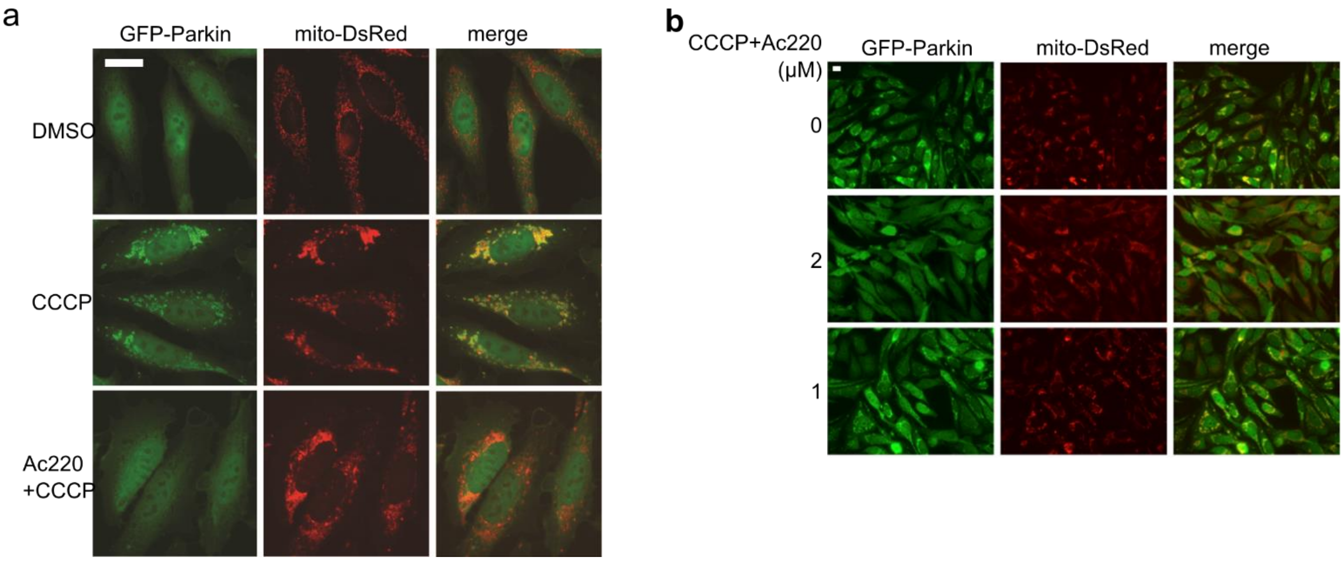

Supplementary Figure 8: Ac220 inhibits Parkin recruitment in a dose-dependent manner. (a) HeLa cells expressing GFP Parkin WT and mito-dsRed treated with either DMSO, 10 µM CCCP or 4 µM Ac220 pre-treatment followed addition of 10 µM CCCP. Representative images displayed, n=3 independent experiments performed with similar results. Scale bars, 10µm. (b) Cells were pre-treated with either DMSO, 2 µM Ac220, 1 µM Ac220 prior to addition of CCCP. Representative images displayed, n=3 independent experiments performed with similar results.

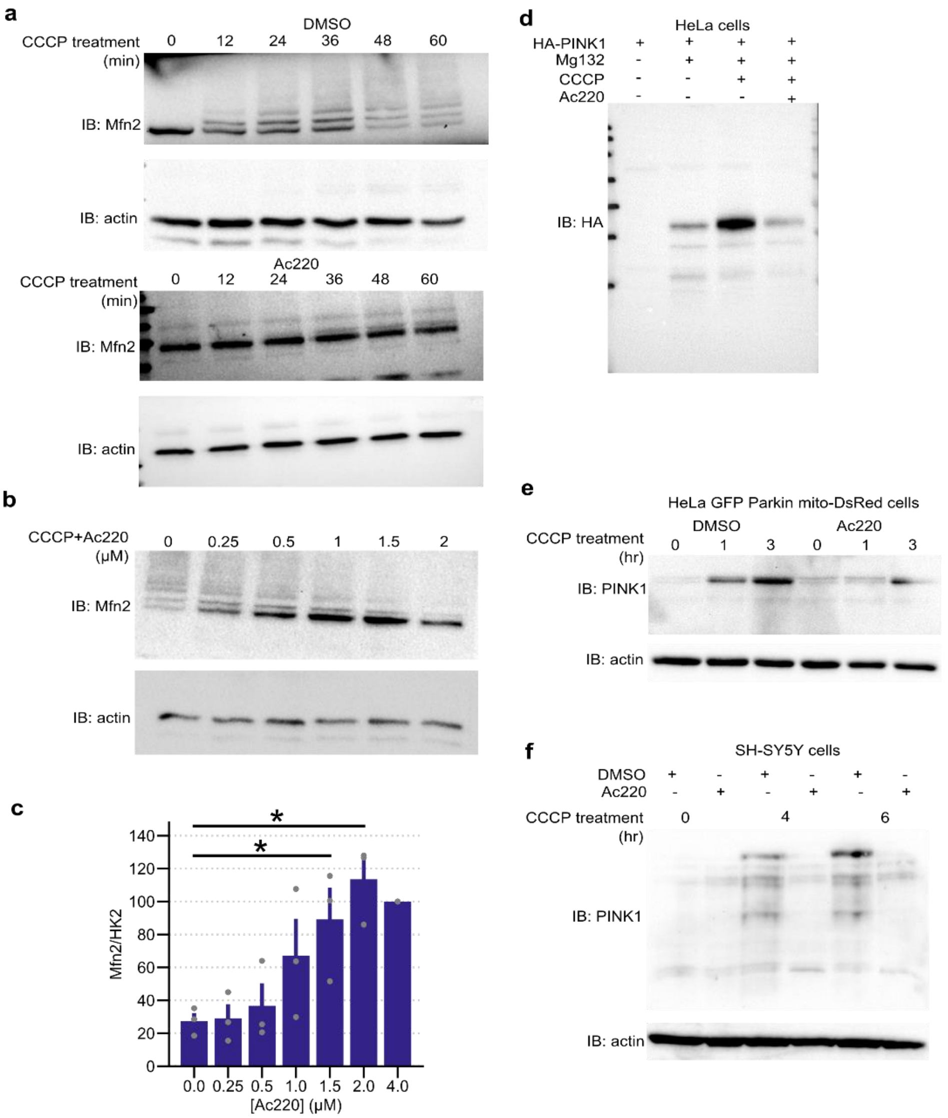

Supplementary Figure 9: Ac220 inhibits PINK1 accumulation and degradation of Mfn2. (a) Cell lysates from HeLa GFP Parkin cells pre-treated with either DMSO or 4 μM Ac220 prior to addition of 10 μM CCCP for the indicated time were separated by SDS-PAGE and immunoblotting was performed against Mfn2 and actin. Representative blots are displayed, n=3 independent experiments were performed with similar results. (b) Cells were treated with varying doses of Ac220 prior to addition of CCCP for 1 hour. Immunoblotting was performed using antibodies against Mfn2 and actin. (c) Densitometry analysis of Mfn2 levels normalized to loading control HK2 (n=3 independent experiments). Data is presented as mean ± s.e.m. P-values were derived from one-tailed paired student's t-test, *P-value<0.05. Error bars represent s.e.m. (d) HeLa GFP Parkin cells were transfected with HA-PINK1 and pre-treated with either DMSO or Ac220 prior to addition of CCCP and Mg132. Immunoblotting was performed with HA antibody. (e) HeLa GFP Parkin cells and (f) SH-SY5Y cells were pre-treated with either DMSO or Ac220 prior to addition of CCCP for the indicated time. Immunoblotting was performed using antibodies against PINK1 and actin. Representative images are displayed for a,d,e and f, n=3 independent experiments were performed with similar results.

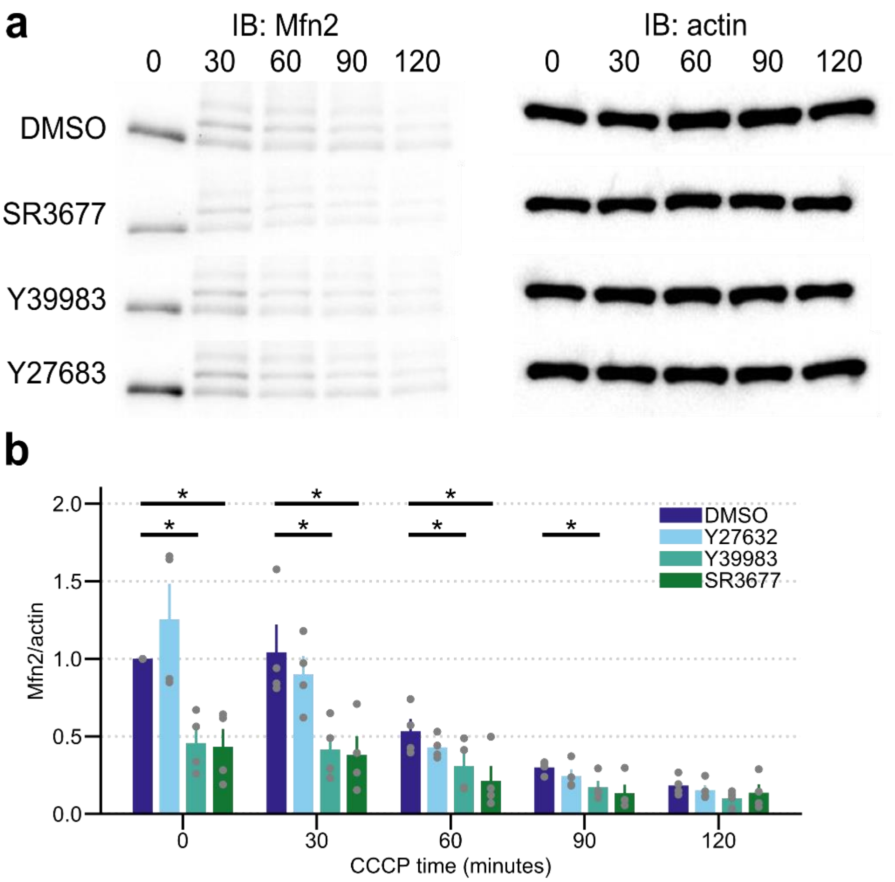

Supplementary Figure 10: Degradation of Mfn2 is increased by ROCK inhibitor treatment. (a) HEK293 GFP Parkin cells were treated with either DMSO, 10 µM Y7632, 10 µM Y39983 or 0.5 µM SR3677 for 2 hours prior to 10 µM CCCP addition. Cell lysates were separated by SDS-PAGE and immunoblotting was performed against Mfn2 and actin. (b) Densitometry quantification of Mfn2 normalized to actin and an internal control within each blot (cells treated with DMSO only (n=4 independent experiments). P-values were determined by paired student's t-test, **P-value*<0.05. Data is expressed as mean ± s.e.m. Error bars represent s.e.m.

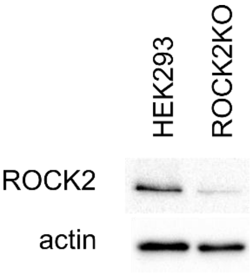

Supplementary Figure 11: Western blot validation of ROCK2 antibody. Cell lysates from HEK293 parental cell line and HEK293 cells following CRISPR-mediated genome editing to knock out ROCK2. Representative blots are displayed, n=3 independent experiments were performed with similar results.

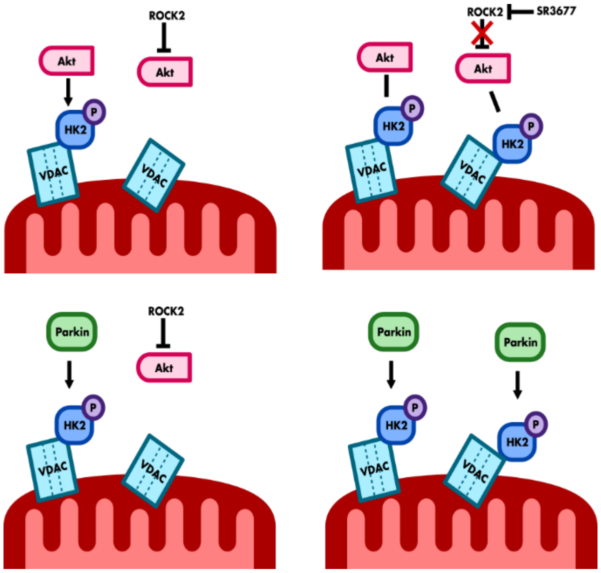

Supplementary Figure 12: The proposed mechanism through which SR3677 increases Parkin recruitment. Hexokinase 2 (HK2) activity is required for recruitment of Parkin to damaged mitochondria. The Akt signaling axis promotes the activation of HK2. ROCK negatively regulates Akt, resulting in the reduction of active HK2 localized to mitochondria. ROCK inhibition releases this brake on Akt signaling, resulting in increased mitochondrial HK2

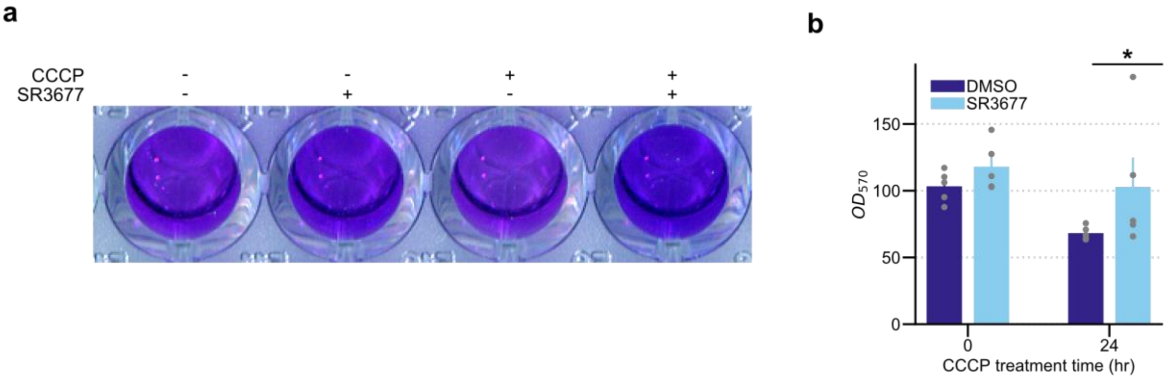

Supplementary Figure 13: SR3677 rescues cell viability loss caused by prolonged CCCP treatment in SH-SY5Y cells. (a) SH-SY5Y cells were pre-treated with either DMSO or 0.5µM SR3677 prior to addition of either DMSO or 10µM CCCP for 24 hours. Following treatment, cells were washed with PBS, fixed with 4% PFA and stained with crystal violet dye. (b) The OD at 570 nm was measured to quantify crystal violet staining (n=4 independent experiments). P-values were determined by paired student's t-test, *P<0.05. Data is expressed as mean ± s.e.m. Error bars represent s.e.m.

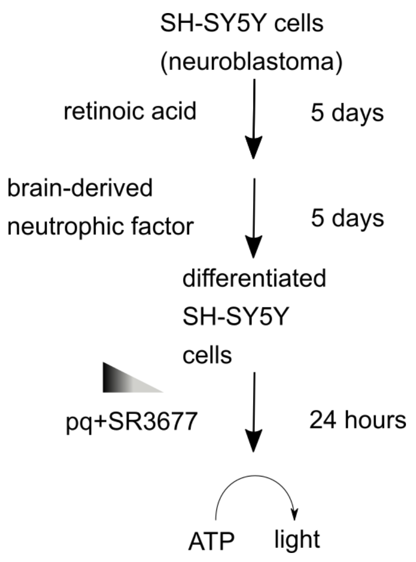

Supplementary Figure 14: Protocol for SH-SY5Y cell differentiation and viability determination workflow.

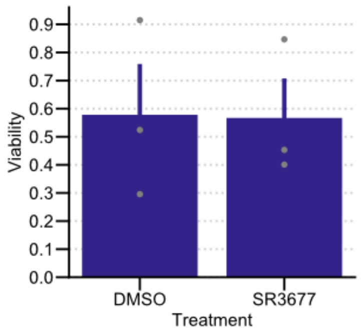

Supplementary Figure 15: SR3677 does not affect the viability of differentiated SH-SY5Y cells. Cell viability (ATP levels normalized to protein concentration per well) of differentiated SH-SY5Y cells treated with DMSO or 4 μM SR3677 (n=3 independent experiments) Data is presented as mean ± s.e.m. Error bars represent s.e.m.

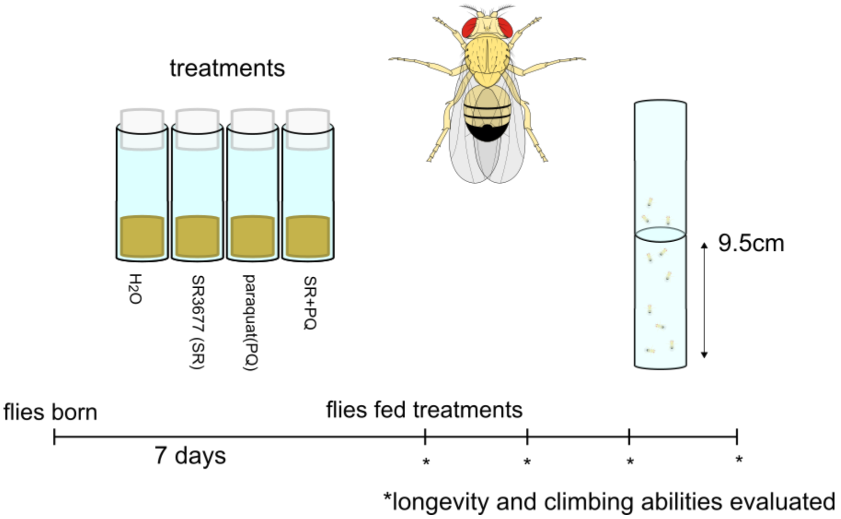

Supplementary Figure 16: Workflow for testing SR3677 in neurotoxin *Drosophila* PD model. Male Canton(S) flies aged 7 days are fed standard yeast, agar and molasses diet supplemented with either water (control), 1 mM SR3677 (SR), 10 mM paraquat (PQ) or SR3677 in combination with paraquat. The survival of flies was recorded each day following administration of the treatments or climbing ability was tested every 2 days by performing negative geotaxis assays.

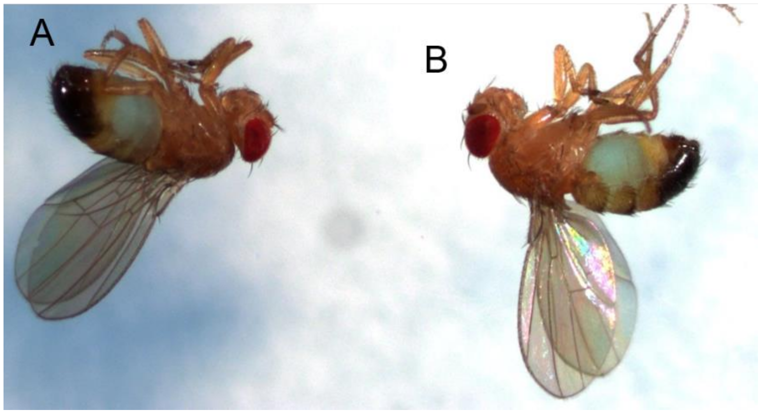

Supplementary Figure 17: Drosophila consume both paraquat and SR3677. Fly food was supplemented with either (A) paraquat or (B) SR3677 along with blue dye FD&C Blue Food Dye No. 1. (n=3 independent experiments).

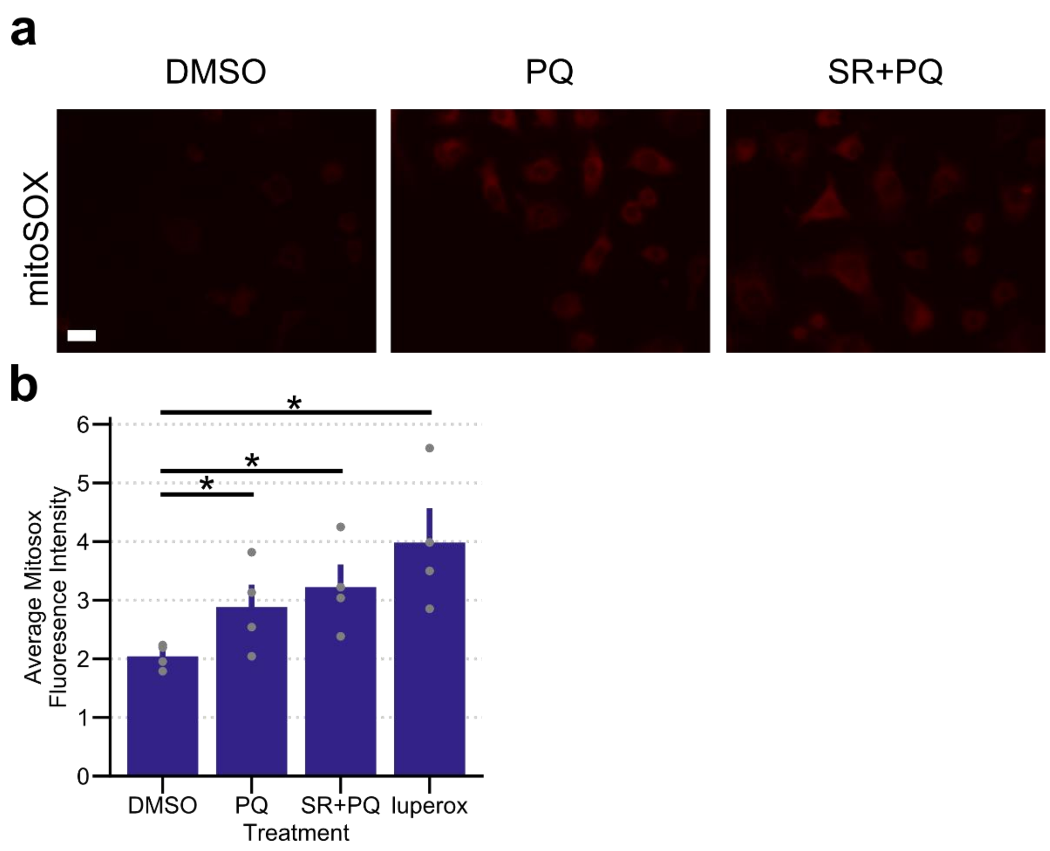

Supplementary Figure 18: SR3677 does not interfere with paraquat-mediated production of mitochondrial reactive oxygen species. S2R+ cells were treated with DMSO, paraquat (PQ), SR3677 in combination with paraquat or the hydroperoxide luperox. Staining was performed with mitoSOX Red Mitochondrial Superoxide dye. Pictured are representative images of cells following the indicated treatments. Scale bars, 10µm. b) Quantification of the fluorescence intensity of the mitoSOX probe following the indicated treatments (n=4 independent experiments). Data is presented as mean ± s.e.m. P-values were determined by paired student's t-test, **P-value*<0.05. Error bars represent s.e.m.

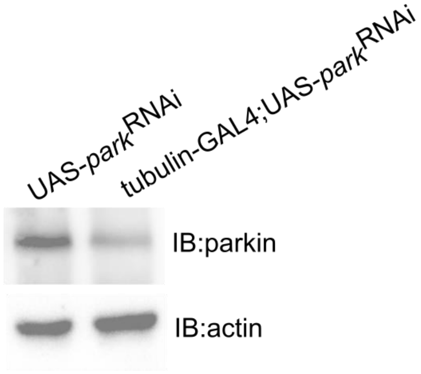

Supplementary Figure 19: RNAi-mediated knockdown of parkin in whole flies. Protein lysates from UAS-park^{RNAi} or tubulin-GAL4;UAS-park^{RNAi} whole flies were separated by SDS-PAGE. Immunoblotting with anti-Parkin and anti-actin antibody was performed. Representative blot is displayed, n=3 independent experiments were performed with similar results.

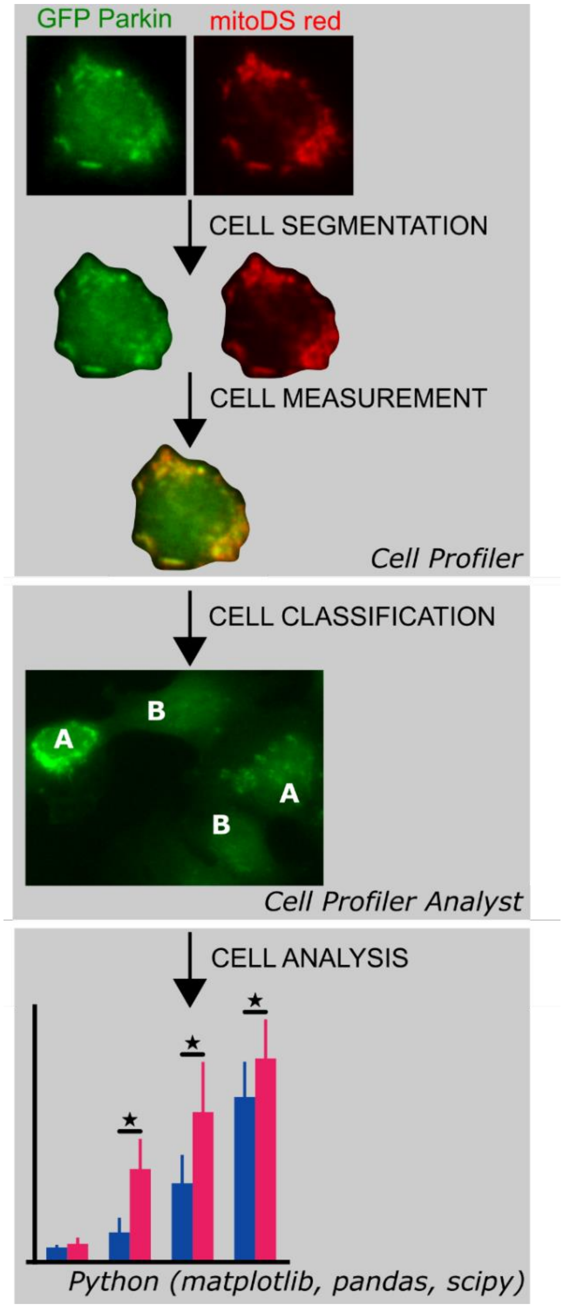

Supplementary Figure 20: Methodology for analysis of Parkin recruitment to damaged mitochondria. Green (GFP-Parkin) and red (mitoDsRed) channel fluorescence microscopy images were collected. Cell Profiler software was used to segment images into areas containing individual cells, to perform feature extraction and image data measurements within each cell area. Cell Profiler Analyst was used to assemble a labelled training set of cells bellowing to two phenotypic subtypes: cells with mitochondrial (A) and cytosolic (B) Parkin distribution and to generate labels for all cells within each image. The data set of labelled cells was subsequently analyzed in Python. Pandas was used for tabular operations, scipy was used for statistical tests, and matplotlib was used to generate visualization. Each bar represents the average percentage of cells with mitochondrial Parkin, the error bars represent the s.e.m. and the * designate P-value<0.05.

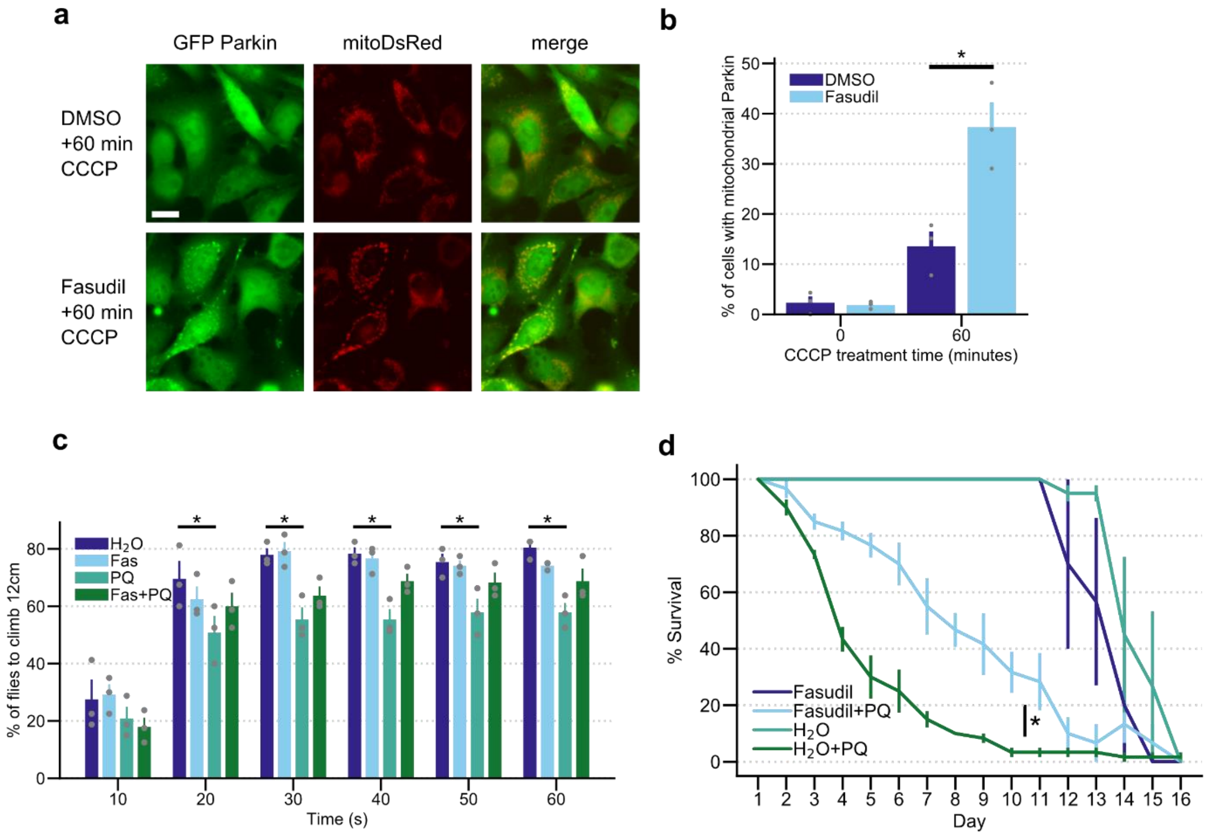

Supplementary Figure 21: Fasudil increases Parkin recruitment to damaged mitochondria, which translates into improved survival and climbing ability in flies treated with paraquat. (a) HeLa cells expressing GFP Parkin and mitoDsRed were pre-treated with 4 µM Fasudil or DMSO for 2 hours prior to addition of either 10 µM CCCP or DMSO for 1 hour. Pictured are representative images of cells following the indicated treatments. Scale bars, 10µm. (b) Quantification of the percentage of cells with mitochondrial Parkin distribution (n=3 independent experiments). (c) 7-day old male Canton(S) flies were placed into vials containing either water, 1 mM Fasudil, 1 mM paraquat (PQ) or paraquat in combination with Fasudil and climbing ability was assessed after 7 days of treatment (n=3 independent experiments). (d) Survival of 7-day old *Drosophila* transferred into fly food vials supplemented with water, 1 mM Fasudil, 5 mM paraquat and paraquat in combination with Fasudil (n=3 independent experiments). P-values were determined by performing log rank sum tests, *P-value*<0.05. Error bars represent s.e.m.

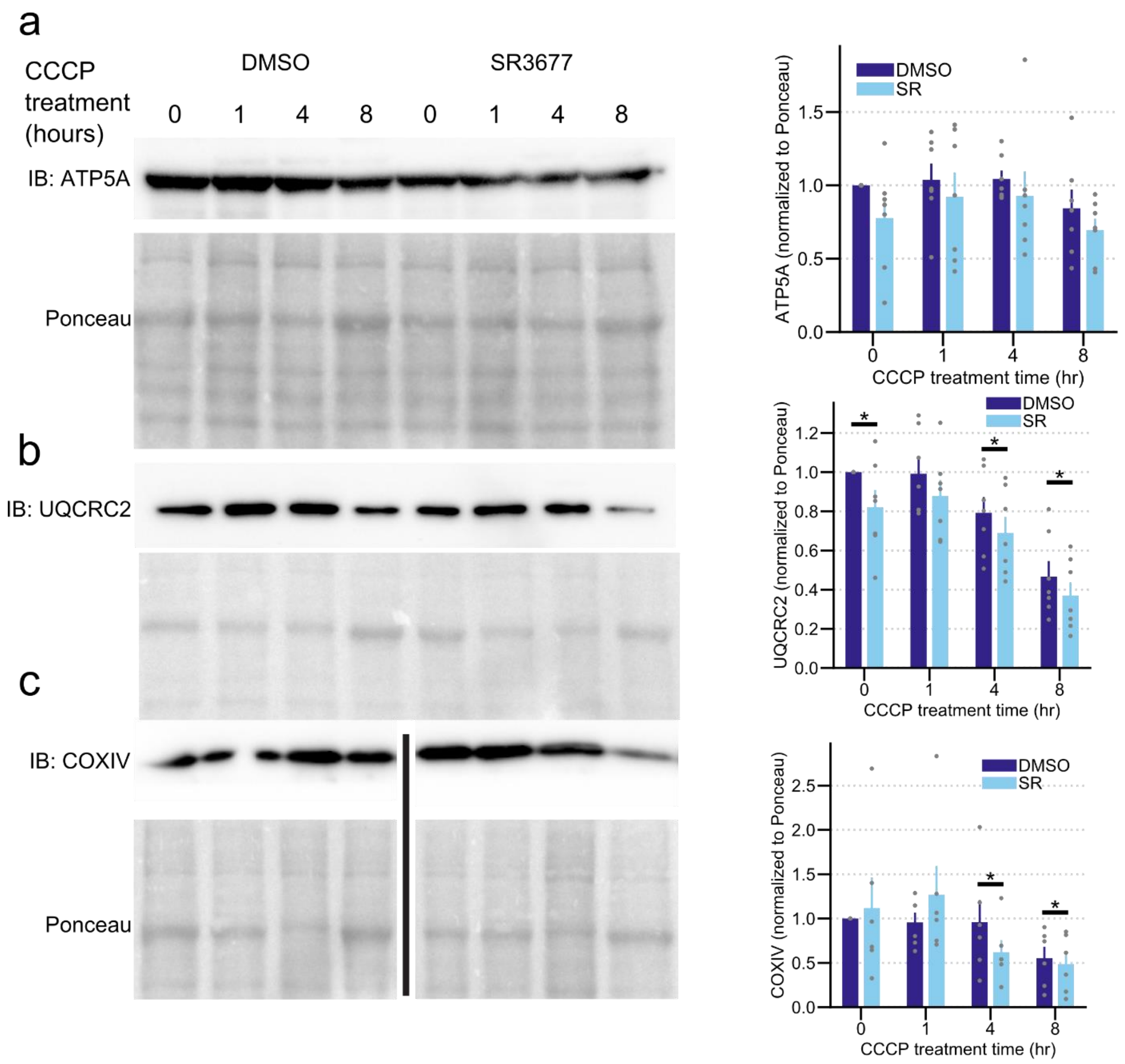

Supplementary Figure 22: SR3677 increases degradation of mitochondrial proteins following induction of mitophagy. HEK293 GFP Parkin cells co-treated with 0.5 µM SR3677 or DMSO and 10 µM CCCP for the indicated time (hours). Cell lysates were separated by SDS-PAGE and immunoblotting was performed to assess turnover of IMM/matrix substrates (a) ATP5A, (b) UQCRC2 and (c) COXIV. Densitometry analysis was performed. Protein levels were normalized to Ponceau. Data is expressed as mean ± s.e.m (n= 6, 7 and 6 independent experiments for a, b and c, respectively). P-values were determined by one-tailed paired student's t-test, *P<0.05. Error bars represent s.e.m.

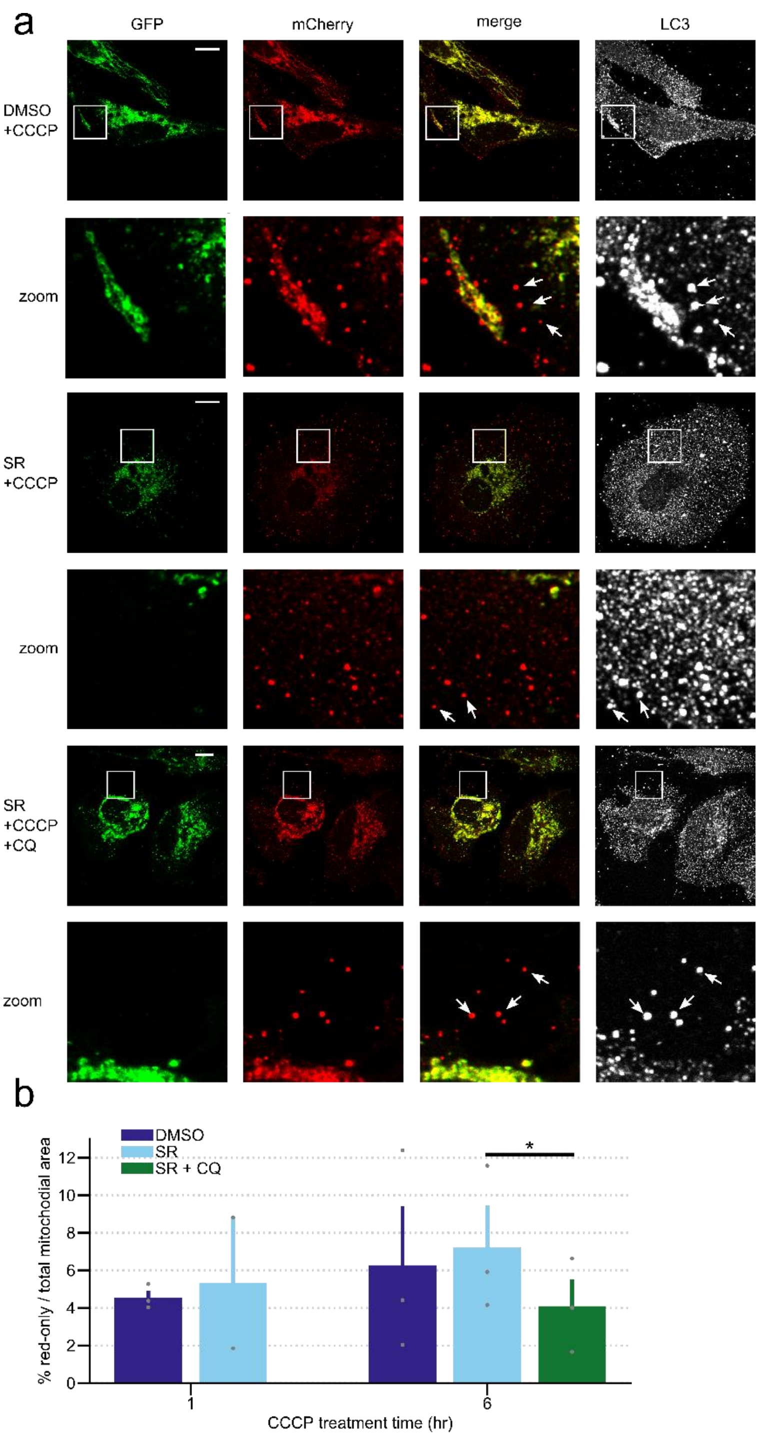

Supplementary Figure 23: Chloroquine abrogates SR-mediated increase to the percentage of mitochondria localized to lysosomes. a) HeLa cells were transfected with Flag-Parkin and RG-OMP25. Following pre-treatment of cells with DMSO or SR3677, CCCP was added to induce mitophagy. Immunostaining against LC3 was performed. Scale bars, 10μm. b) Quantification of the percentage of red-only mitochondrial area corresponding to mitochondria localized to lysosomes. Data is expressed as mean ± s.e.m (n=3 independent experiments). P-values were determined by one-tailed paired student's t-test, **P*<0.05. Error bars represent s.e.m.

Fig. 2

a

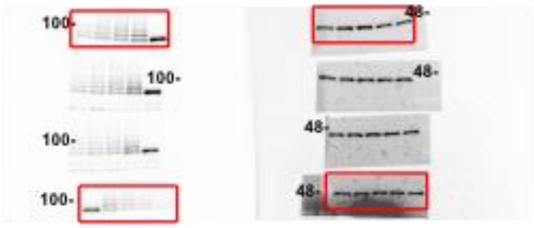

IB: Mfn2 IB: actin

c

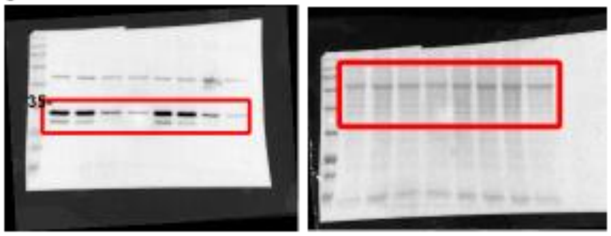

IB: VDAC1 Ponceau

Fig. 4

b

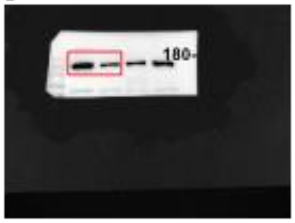

IB: ROCK2

e

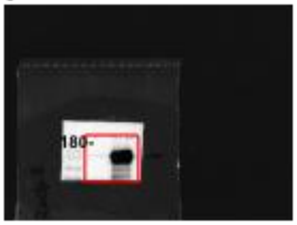

IB: ROCK2

Fig. 5

c

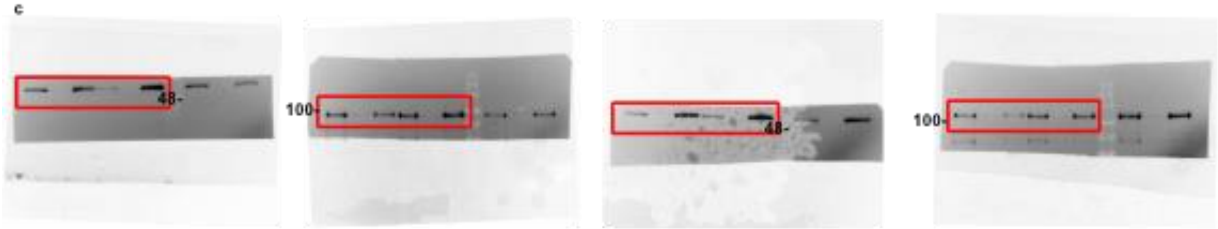

IB: CValpha IB: HK2 IB: CValpha IB: HK2

e

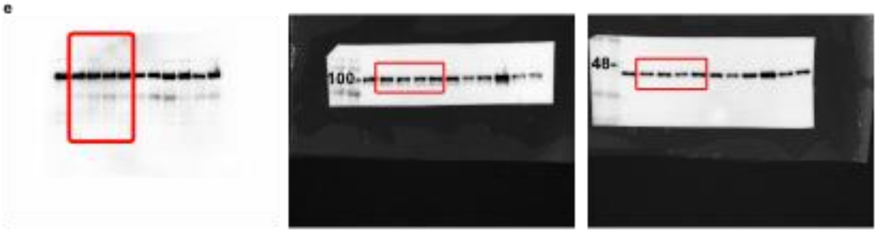

Phos-tag IB: HK2 IB: HK2 IB: actin

Supplementary Fig. 2

c

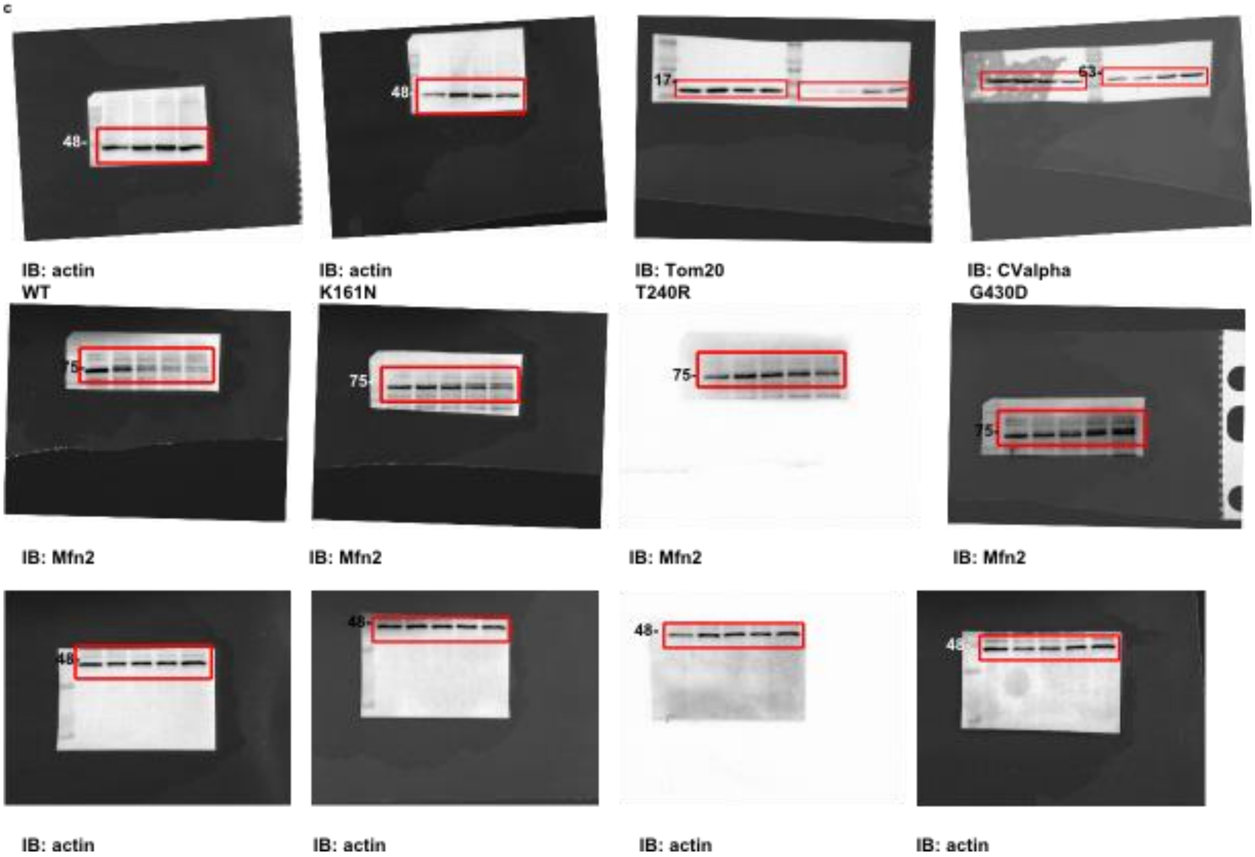

Supplementary Fig. 10a

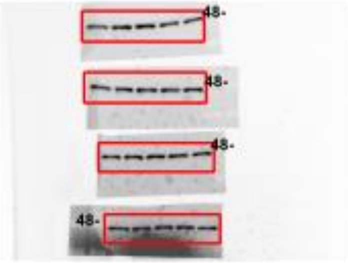

IB: actin

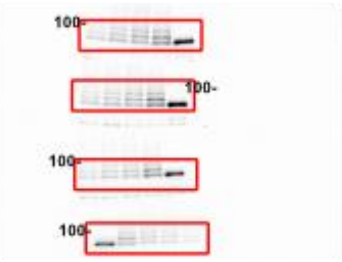

IB: Mfn2

Supplementary Fig. 11

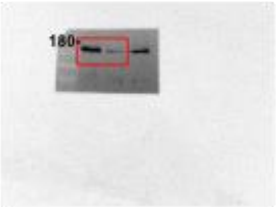

IB: ROCK2

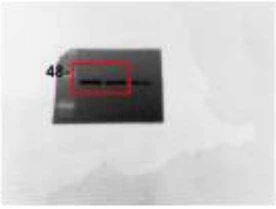

IB: actin

Supplementary Fig. 19

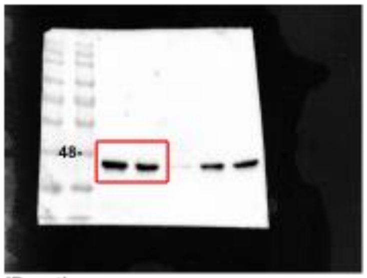

IB: actin

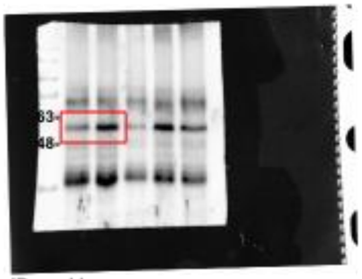

IB: parkin

Supplementary Fig. 22

a

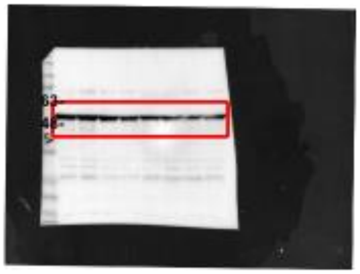

IB: ATP5A

b

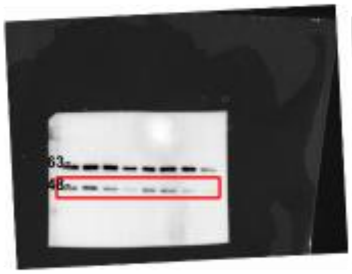

IB: UQCRC2

c

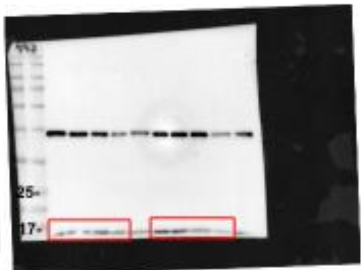

IB: COXIV

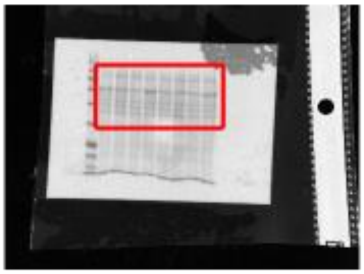

Ponceau

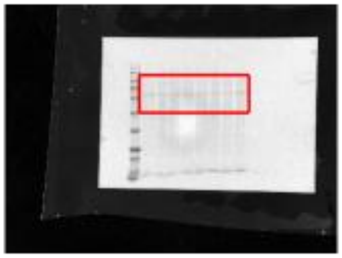

Ponceau

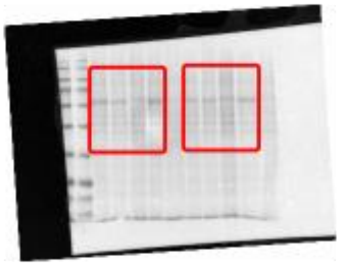

Ponceau
