# Supplementary material for: ROCK inhibitors upregulate the neuroprotective Parkin-mediated mitophagy pathway
Source: Nat Commun. 2020 Jan 3;11:88. doi: 10.1038/s41467-019-13781-3 (PMC6941965; doi:10.1038/s41467-019-13781-3)
Supplement: Supplementary file 2 — Description of Additional Supplementary Files [file 41467_2019_13781_MOESM2_ESM.docx]

Description of Additional Supplementary Files

**File name**: Supplementary_Data

**Description**: Screening data corresponding to Figure 1. Table displays the average activity (% of cells with mitochondrial Parkin) and z-score (xcompound-uscreen/ across two independent experiments for each compound screened.
